# Activation of integrin signaling up-regulates pro-inflammatory cytokines in JAK2-V617F positive hematopoietic cells

**DOI:** 10.1186/s12964-025-02358-x

**Published:** 2025-08-11

**Authors:** Conny K. Baldauf, Corinna Fahldieck, Alexa Angenstein, Sönke Weinert, Mariam Hakobyan, Daniel B. Lipka, Tobias R. Haage, Vikas Bhuria, Martin Böttcher, Dimitrios Mougiakakos, Burkhart Schraven, Thomas Fischer

**Affiliations:** 1 Institute for Molecular and Clinical Immunology, Medical Faculty, Otto-von-Guericke University, Magdeburg, Germany; 2 Healthcampus Immunology, Inflammation and Infectiology (GC-I3), Medical Faculty, Otto-von-Guericke University, Magdeburg, Germany; 3 Department of Hematology, Oncology and Cell Therapy, Medical Faculty, Otto-von-Guericke University, Magdeburg, Germany; 4 University Clinic for Cardiology and Angiology, Otto-von-Guericke University, Medical Faculty, Magdeburg, Germany; 5 Section of Translational Cancer Epigenomics, Division of Translational Medical Oncology, German Cancer Research Center (DKFZ), Heidelberg, Germany; 6 National Center for Tumor Diseases (NCT), NCT Heidelberg, a partnership between, DKFZ and Heidelberg University Hospital , Heidelberg, Germany; 7 German Cancer Consortium (DKTK), Heidelberg, Germany; 8 Center for Health and Medical Prevention - CHAMP, Otto-von-Guericke University, Magdeburg, Germany

**Keywords:** Integrins, Adhesion, VCAM-1, ICAM-1, Pro-inflammatory cytokines, JAK2-V617F, MPN, Inflammation, IL-1α, IL-1β

## Abstract

**Background:**

The JAK2-V617F mutation is the most frequent driver mutation in a group of malignant hematopoietic disorders called myeloproliferative neoplasms (MPN). JAK2-V617F is a somatic mutation originating in a hematopoietic stem cell and results in constitutively activated JAK-STAT signaling. High levels of pro-inflammatory cytokines in the blood are a hallmark of MPN patients and are a key factor in the severe clinical symptoms seen in these patients. The molecular mechanisms underlying the up-regulation of inflammatory cytokines in JAK2-V617F mutated hematopoietic cells remain to be elucidated.

**Methods:**

32D myeloid progenitor cells expressing JAK2-wildtype (WT) and JAK2-V617F, respectively were employed. In addition, primary hematopoietic cells from the JAK2-V617F knock-in MPN mouse model were investigated. Integrin outside-in signaling upon binding of cells to the adhesion molecules VCAM-1/ICAM-1 was characterized by Western blotting of phosphorylated FAK, STAT3, p65, SYK and JNK. Regulation of mRNA and protein expression of IL-1α, IL-1β, IL-6, TNF and CXCL10 was measured by qPCR and ELISA. RNAseq and DNA methylation analysis in primary mouse JAK2-V617F granulocytes was performed. In JAK2-V617F knock-in mice, anti-integrin treatment was applied to evaluate the impact of activated integrin signaling on IL-1 blood levels in vivo.

**Results:**

Integrin stimulation via the adhesion molecules VCAM-1/ICAM-1 activated integrin outside-in signaling including FAK, SYK, NFκB, and JNK. This induced strong mRNA expression of IL-1α, IL-1β, IL-6, TNF and CXCL10. In 32D cells, the presence of the JAK2-V617F mutation further increased VCAM-1/ICAM-1-induced mRNA and protein levels of IL-1α and IL-1β, and active caspase 1 expression. In primary granulocytes, integrin stimulation resulted in an activated mRNA signature of inflammatory cytokines. Consistent with the mRNA results, adhesion to VCAM-1/ICAM-1 induced an increase in intracellular IL-1α and IL-1β protein levels in 32D cells. However, in primary hematopoietic cells, up-regulation of inflammatory cytokines was not observed at the protein level in vitro, whereas, in vivo, blocking of integrin binding to VCAM-1/ICAM-1 was sufficient to reduce elevated IL-1α levels in the blood of JAK2-V617F mice.

**Conclusions:**

We conclude that integrin stimulation via the adhesion molecules VCAM-1/ICAM-1 activates integrin outside-in signaling, leading to the up-regulation of pro-inflammatory cytokines in both JAK2-mutated and non-mutated mouse hematopoietic cells.

**Supplementary Information:**

The online version contains supplementary material available at 10.1186/s12964-025-02358-x.

Partly presented in abstract form at the 64th annual meeting of the American Society of Hematology, December 2023 [1]. In partial fulfillment of the PhD thesis of C.K.B.

## Introduction

Myeloproliferative neoplasms (MPN) are malignant clonal hematopoietic disorders which are driven by somatic mutations in the hematopoietic stem cell compartment. This leads to abnormal proliferation of myeloid cells. The main driver mutations are found in JAK2, Calreticulin and MPL [2]. The classic myeloproliferative neoplasms (MPNs) include diseases such as polycythemia vera (PV), essential thrombocythemia (ET) and primary myelofibrosis (PMF) [2, 3]. PV and ET are primarily characterized by a strong increase in the erythrocyte and platelet (thrombocyte) count, respectively [2, 3]. MF presents with increasing bone marrow fibrosis and increased spleen size (splenomegaly) [2, 3]. The most common mutation in MPN is the JAK2-V617F (JAK2-VF) mutation [4–7], which constitutively activates JAK-STAT signaling [8–10]. In the presence of a cytokine receptor such as the erythropoietin receptor (EPOR), the JAK2-VF kinase induces growth-factor independent proliferation and resistance to apoptosis in hematopoietic cells [11]. JAK2-VF also activates β1 and β2 integrins as demonstrated for VLA-4 and LFA-1 in human and mouse granulocytes [12, 13]. Integrins play a pivotal role in communication and adhesion of hematopoietic cells and in inflammatory conditions. They are bidirectional adhesion molecules that are normally expressed in a bent and inactive conformation [14]. Integrins are transmembrane heterodimers, each consisting of one α-subunit and one β-subunit. JAK2-VF constitutively activates β1 and β2 integrins via activation of “**in**side-out signaling” of integrin receptors [12, 13]. Thus, JAK2-VF switches on the small GTPase Rap1 in a calcium dependent manner via CalDAG-GEFI and PI3K [13]. In JAK2-VF-positive granulocytes, Rap1 is constitutively active and translocates from the cytosol to the inner leaflet of the cell membrane thereby attaching to the cytoplasmic domains of β1 and β1 integrin receptors [13]. Upon activation of inside-out signaling, integrins turn into an open and active conformation and bind their ligands with high affinity [14]. In JAK2-VF positive cells, this results in increased binding to the major adhesion molecules VCAM-1 and ICAM-1 [12, 13]. As a consequence, **out**side-in signaling is activated which involves a variety of signaling molecules. Their activation is dependent on the particular stimulus, the specific integrin, the integrin ligand and the cell type [15–17]. In this study, we investigated the major leukocyte-specific integrins VLA4 (α4β1) and the β2 integrins interacting with VCAM-1 [18] and ICAM-1, respectively. Integrin β2 (= CD18) is the β chain of four heterodimers: α_L_β_2_ (LFA-1), α_M_β_2_ (Mac-1), α_X_β_2_ and α_D_β_2_ [15]. VCAM-1 and ICAM-1 are expressed on endothelial cells, stromal cells and others [19]. In inflammation, their membrane expression is highly up-regulated [20, 21].

An important clinical feature of MPN is a pronounced inflammatory state which is associated with highly elevated pro-inflammatory cytokine levels (cytokine storm) in the blood of JAK2-VF-positive MPN patients [22–26]. High inflammatory cytokines in patients are associated with harmful clinical symptoms as fever, night-sweats, cachexia and generally result in poor quality of life [27]. The cellular and molecular events involved in generation of the pro-inflammatory cytokine signature in the blood of MPN patients are poorly understood. The approval of JAK1/2-inhibitors (e.g. Ruxolitinib) for therapy of MPN patients has been shown to reduce pro-inflammatory cytokine levels and to improve the quality of life in patients.[27].

In recent years, the pivotal role of interleukin-1 (IL-1) in MPN pathogenesis has been increasingly recognized [28–31]. IL-1 promotes clonal expansion of JAK2-VF mutated hematopoietic stem and progenitor cells (HPSC) [30]. IL-1 also promotes disease progression in mouse models by increasing bone marrow fibrosis and damage to sympathetic nerve fibres in the stem cell niche [28–31]. IL-1 is a major regulator of inflammation and is primarily represented by two subtypes: IL-1α and IL-1β. Knock-out of IL-1β in hematopoietic cells of JAK2-VF MPN mice was shown to reduce inflammatory cytokines [28]. Both IL-1α and IL-1β, are pro-inflammatory, pyrogenic, and pleiotropic [32]. Elevated IL-1 serum cytokine concentrations have been linked to a number of malignant and autoimmune disorders [33, 34]. IL-1α and IL-1β are expressed as 31 kDa pro-forms [35] devoid of a signal peptide, which is necessary for Golgi-dependent classical secretion [36]. In contrast to pro-IL-1β, pro-IL-1α is already biologically active [37]. Different cleavage sites of the pro-forms result in different maturation pathways [38]. Two signals are required for the expression and maturation of IL-1β: a priming signal and an activation signal for the inflammasome (e.g. NLRP3). Once the NLRP3 inflammasome is activated, caspase 1 is cleaved [39]. The activated caspase 1 is then able to cleave pro-IL-1β into its mature and biological active form [38, 40]. Active caspase 1 also cleaves gasdermin D [41], thereby inducing pore formation [42]. The cell death induced in this way is called pyroptosis [41, 42]. Mature IL-1β is typically passively secreted by pyroptosis. Conversely, pro-IL-1α is cleaved by different enzymes (e.g. calpain, thrombin) and exhibits different localizations within the cell. The N-terminus of the protein and the pro-form are capable of entering the nucleus and is able to activate transcription [43]. IL-1α is also able to translocate to the cell membrane, where it induces local immune responses [32, 44]. In contrast, the mature and secreted IL-1α induces a systemic immune response [45].

Using in vitro and in vivo experimentation, we here sought to test the hypothesis that activation of integrin β1 and β2 receptors via their natural ligands VCAM-1 and ICAM-1 induces pro-inflammatory cytokines in JAK2-VF positive cells.

## Methods

### Cell culture

Murine myeloid progenitor 32D cells, expressing the JAK2-V617F mutation (JAK2-VF cells) or the JAK2-wild type (WT) as previously described [12, 13, 46] were used. The 32D JAK2-WT and 32D JAK2-VF cell lines were stably transfected with the erythropoietin receptor (EPOR) and cultured in RPMI 1640 medium supplemented with 10% FCS and 25 U/ml penicillin/streptomycin as previously reported [13, 46]. 32D JAK2-WT/EPOR and 32D JAK2-VF/EPOR cells were indicated as 32D JAK2-WT and 32D JAK2-VF in the main text and in the legends to the figures to facilitate reading. For maintaining cell growth, 1 I.U. EPO/ml was added to the 32D JAK2-WT/EPOR cells. 32D JAK2-VF/EPOR cells proliferate growth-factor independently. For starvation experiments, serum-reduced (0.5%) medium was used without EPO.

### JAK2-VF mouse model and anti-VLA4/β2-integrin treatment

*Vav1-Cre x Jak2*^+*/*+^ or *Vav1-Cre x Jak2*^*VF/*+^ mice have been described previously [13, 47]. Physiological expression of JAK2-VF in *Vav1-Cre x Jak2*^*VF/*+^ mice causes a lethal MPN-like disease. Bone marrow was isolated from *Jak2*^*VF/*+^ or *Jak2*^+*/*+^ mice following standard procedures. Isolation of granulocytes (negative isolation) was performed by MACS using streptavidin BD IMag™ Particles Plus – DM (BD Bioscience) and biotin-labeled antibodies. anti-CD5 (clone 53–7.3), anti-B220 (clone RA3-6B2), anti-c-Kit (clone 2B8), anti-F4/80 (clone BM8) and anti-Terr119 (clone Ter119) were purchased from Biolegend and anti-CD49b (clone HMa2) from ebioscience. The purity of the granulocytes post-isolation was determined to be 87.3% ± 1.7%.

*Vav1-Cre x Jak2*^+*/*+^ and *Jak2*^*VF/*+^ control mice (10-weeks old) were administered a single intraperitoneal injection of PBS (200 μl) on day 1. *Vav1-Cre x Jak2*^*VF/*+^ mice were injected intraperitoneally with 200 µg each of anti-VLA4 and anti-β2 integrin (total 200 μl) antibodies (clone PS/2, BioXCell; clone GAME-46, BD Pharmingen™) or the corresponding IgG isotype control antibodies (200 µg of each isotype control; total 200 μl) on day 1 (clone LTF-2, BioXCell; clone R3-34, BD Pharmingen™). The antibody clones used, the route of administration and the dose of anti-integrin antibodies have been described previously [13]. On day 8 post-injection, blood was collected for serum cytokine analysis, which was conducted by EveTechnologies (Calgary, AB Canada) using the Mouse Cytokine 32-Plex Assay.

### Western blot analysis

Western Blot samples were generated using 32D JAK2-WT and JAK2-VF cells or isolated total bone marrow cells of *Vav1-Cre x Jak2*^+*/*+^ or *Vav1-Cre x Jak2*^*VF/*+^ mice. The cells were starved for 2 h prior to stimulation for 15 min by immobilized VCAM-1 and/or ICAM-1. BSA was utilized as a control (unstimulated cells). Therefore, 24-well plates were coated overnight at 4 °C with 3 µg/ml recombinant murine VCAM-1 (V125, Leinco Technologies) and/or ICAM-1 (I-587, Leinco Technologies) or 1% BSA. In some experiments as indicated, 32D JAK2-VF cells were pre-incubated with Fc-Block (TruStain FcX™ PLUS antibody; Biolegend) for 15 min as well as anti-VLA4/β2-integrin antibodies or the corresponding IgG isotype control (BioXCell, BD Pharmingen™). Signaling molecule inhibitors (Supplementary Table 1) were applied for 15 min before cells were co-stimulated with immobilized VCAM-1/ICAM-1. Cells were directly lysed with supplemented lysis buffer [12, 13] in the plate. Western Blot was conducted as previously described [13]. The antibodies used are listed in Supplementary Table 2.

### qPCR analysis

qPCR samples were generated using 32D JAK2-WT and JAK2-VF cells, isolated total bone marrow cells or isolated granulocytes from *Vav1-Cre x Jak2*^+*/*+^ or *Vav1-Cre x Jak2*^*VF/*+^ mice. Prior to stimulation for 1 h (primary cells) or 3 h (32D cells) at immobilized or soluble VCAM-1 and/or ICAM-1 (c = 3 µg/ml), the cells were starved for 2 h. BSA was used as an unstimulated control. In experiments indicated, 32D JAK2-VF cells were pre-incubated with Fc-block (TruStain FcX™ PLUS antibody; Biolegend) for 10 min as well as anti-VLA4/β2-integrin antibodies or the corresponding IgG isotype control for 15 min. Signaling molecule inhibitors (Supplementary Table 1) or commonly used clinical drugs (Ruxolitinib, Hydroxyurea; Supplementary Table 1) were applied for 15 min before co-stimulation with immobilized VCAM-1/ICAM-1. Following stimulation, the supernatant was removed, the cells were washed with HBSS (Gibco), and the adherent cells were lysated in Trizol (Ambion®) and pooled with the cells from the supernatant. Isolation of RNA was conducted in accordance with the manufacturing protocol. Cytokine mRNA was detected by qPCR. RNA was translated into cDNA using the High Capacity cDNA Reverse Transcription Kit (AppliedBiosystems™) and Thermocycler peqSTAR (VWR Peqlab). Subsequently, the qPCR PowerUp™ SYBER™ Green Master Mixes (AppliedBiosystems™) and a QuantStudio 3 Real-Time PCR System (Applied Biosystems™) were employed. The primers are listed in Supplementary Table 3.

### RNA-sequencing

For RNA sequencing, granulocytes were isolated from the bone marrow of *Vav1-Cre x Jak2*^+*/*+^ or *Vav1-Cre x Jak2*^*VF/*+^ mice by MACS, as previously described [13]. The granulocytes were then stimulated for 1 h with immobilized VCAM-1/ICAM-1. RNeasy Mini Kit (Qiagen) was used to extract RNA. RNA sequencing was performed by Genewiz® from Azenta Life Science (GENEWIZ Germany GmbH). The Gene Set Enrichment Analysis (GSEA) was conducted using 1000 permutations, employing the database m5.go.mf.v2024.1.Mm.symbols.gmt and the chip platform Mouse_Ensembl_Gene_ID_MSigDB.v2024.1.Mm.chip for the analysis (GSEA (v4.3.2). For GSEA analysis transcripts per million (TPM) results of the RNA sequencing was used. The R package DESeq2 from BiocManager and package gplots was utilized for analysis. Principal component analysis (PCA) was conducted using R (v4.2.2) and RStudio (v2022.12.0). DESeq2 results of all analyzed genes (TPM) were used for PCA.

### Methylation array

The DNA methylation analysis was performed on isolated and stimulated bone marrow granulocytes derived from *Vav1-Cre x Jak2*^+*/*+^ or *Vav1-Cre x Jak2*^*VF/*+^ mice. Granulocytes were isolated by MACS, as previously described [13]. DNA was isolated using the DNA-Isolation (blood) Kit (Qiagen). A total of 800 ng of DNA of each sample was analyzed using the Infinium Mouse Methylation BeadChip (MMBC) Array (Illumina). This platform interrogates 285,000 methylation sites. The R package RnBeads was utilized for analysis (10.1038/nmeth.3115). Briefly, IDAT files were imported in RnBeads and quality control was performed to examine data quality. Background noise and sex chromosomes were removed along with unreliable probes with by setting *Greedycut p*-value threshold to 0.01. Intra-sample normalization was performed with the method “scaling”. To annotate CpGs to corresponding genes, the Infinium Mouse Methylation Manifest file was acquired from Illumina. DNA methylation data set was subsetted based on CpGs corresponding to genes depicted in Fig. 5C. Hierarchical clustering was performed using DNA methylation of corresponding genes by using the R package ComplexHeatmap and Manhattan distance.

### ELISA measurements

32D JAK2-WT and 32D JAK2-VF cells as well as isolated bone marrow granulocytes of *Vav1-Cre x Jak2*^+*/*+^ or *Vav1-Cre x Jak2*^*VF/*+^ mice (1 × 10^6^ cells) were stimulated for 6 h with immobilized VCAM-1/ICAM-1 or BSA. In some samples indicated, 100 ng/ml LPS (Sigma Aldrich) and/or 3 mM ATP (Sigma Aldrich) were added. In 32D cells, the effects of 5 µM PP2 (Selleckchem) and/or 25 µM Y15 (Selleckchem) were examined. After 6 h incubation, the supernatant was collected and volume-reduced by centrifuge tubes (ROTI®Spin, MINI-3, ROTH; Amicon® Ultra Centrifugal Filter, 3 kDa MWCO, Merck). The cells were lysed directly in the plate with 100 µl (for anti-IL-1β detection) and 200 µl (for anti-IL-1α detection) of PBS + 1% Triton-X-100 (Serva) and Complete (Sigma) for 30 min on ice.

The concentration of mature IL-1α and IL-1β was determined by anti-IL-1 ELISAs according manufacturer´s instructions (IL-1 alpha Mouse Uncoated ELISA Kit with Plates, IL-1 beta Mouse uncoated ELISA Kit with Plates; Invitrogen). The samples (100 µl) were incubated overnight at 4 °C prior to quantification.

### Integrin cluster formation

To investigate the formation of CD29 (β1-integrin) clusters, 32D JAK2-WT and 32D JAK2-VF cells were starved for 2 h prior to stimulation with immobilized VCAM-1/ICAM-1 for 15 min (30,000 cells/70 µl). Therefore, Culture-Insert 2 Well in μ-Dishes 35 mm (ibidi®) was coated overnight with 70 μl of 3 μg/ml mVCAM-1/mICAM-1. Following the stimulation period, the cells were fixed using formaldehyde for 20 min at RT. Then, inserts were carefully removed. μ-Dishes were then washed with PBS. The fixed cells were stained with 200 µl PBS + 10% FCS and 10 µg/ml anti-CD29-AlexaFluor488® antibody (clone HMβ1-1, Biolegend) overnight at 4 °C. Following a washing step, an additional secondary staining was performed using 200 μl PBS + 10% FCS and 1:200 anti-Hamster-AlexaFluor488® (Jackson ImmunoResearch Europe Ltd). Finally, stained and washed cells were covered with a mounting medium and a coverslip. For fluorescent microscopy, a Zeiss Axiovert 200 M (Supplementary Table 4) and a magnification of 63 × was used. For analysis CellProfiler 4.2.1 software was utilized. The analysis is described in detail in Additional file 1.

### IL-1α surface expression

32D JAK2-WT and 32D JAK2-VF cells were stimulated with immobilized VCAM-1/ICAM-1 or BSA for 24 h. Harvested cells were washed, incubated with Fc-block (TruStain FcX™ PLUS antibody; Biolegend) for 10 min on ice and with anti-IL-1α-PE (clone ALF-161, Biolegend) (1:100) for 15 min at room temperature. Following a PBS + 1% FCS washing step, IL-1α surface expression was quantified by flow cytometry (Cytek® Northern Lights™).

### Fluorescent labeled inhibitor of caspases (FLICA) assay

IL-1β is primarily secreted by pyroptosis, which is characterized by active caspases and the formation of pores [48]. Active caspase 1 was detected using FLICA assay (FAM-YVAD-FMK, ImmunoChemistry Technologies) according to the manufacture description. 100,000 32D JAK2-WT and 32D JAK2-VF cells were stimulated in 300 µl supplemented RPMI medium on immobilized VCAM-1/ICAM-1 or BSA for 6 h, respectively. As a positive control, LPS (100 ng/ml) and Nigericin (5 µM) were used, which are known to activate pyroptosis [49]. 1 h before stimulation was terminated, 1:100 30 × FLICA was added. After harvesting and a washing step, the cells were stained with SYTOX™ Blue (1:3000) and directly measured by FACS (Cytek® Northern Lights™). The mean fluorescence intensity (MFI) of FLICA and the percent of FLICA-positive cells were measured. Cells that were double positive for FLICA and SYTOX™ Blue were identified as pyroptotic cells.

### Statistics

Statistical analysis was carried out using GraphPad Prism Version 10 (GraphPad Software Inc., La Jolla, CA, USA) based on number of comparisons and normality distribution. Data are shown as mean ± SEM. The tests used are mentioned in the figure legends. *P*-values below 0.05 were considered significant. *P < 0.05, **P < 0.01; ***P < 0.001, ****P < 0.0001.

## Results

### Stimulation of JAK2-V617F positive hematopoietic cells by VCAM-1/ICAM-1 induces mRNA expression of inflammatory cytokines in a cell-type specific manner

**Fig. 1:**
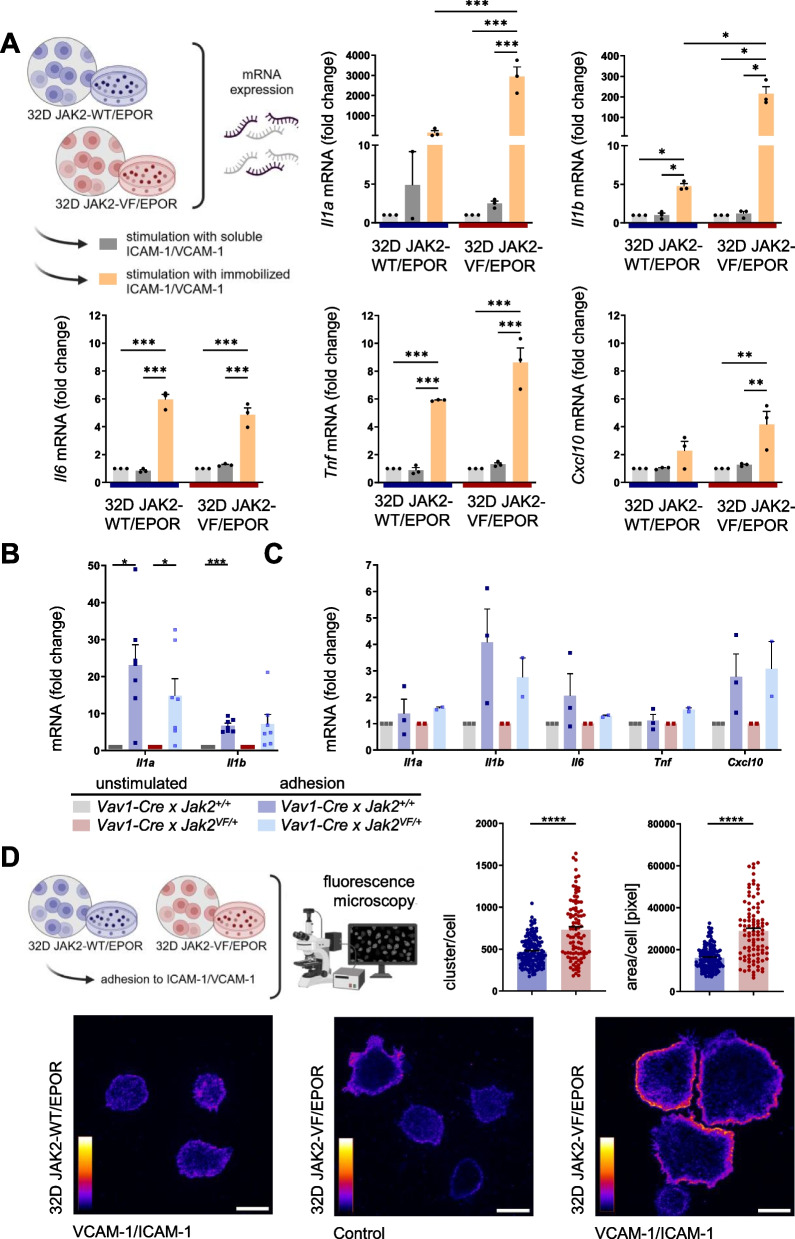
Adhesion to VCAM-1/ICAM-1 up-regulates mRNA expression of inflammatory cytokines. **A** Stimulation (3 h) with immobilized (orange) but not with soluble (dark grey) VCAM-1/ICAM-1 induces mRNA expression of *Il1a, Il1b, Il6, Tnf* and *Cxcl10* in 32D JAK2-WT (left; *n* = 3) and 32D JAK2-VF (right; *n* = 3) cells in comparison to BSA (light grey). Data are shown as fold change (2^−ΔΔCT^) with mean + SEM. 2-Way ANOVA with Tukey’s multiple comparison test P* < 0.05, P** < 0.01, P*** < 0.001. Cartoon depicting the experimental design, created with *Biorender.com (*https://BioRender.com/d34c738*)*. **B** *Il1a* and *Il1b* mRNA expression upon VCAM-1/ICAM-1 stimulation (1 h) using isolated granulocytes of *Vav1-Cre x Jak2*^+*/*+^ (*n* = 7) and *Vav1-Cre x Jak2*^*VF/*+^ mice (*n* = 7) is shown as fold change compared to the BSA control (“unstimulated”). Data are shown as fold change (2^−ΔΔCT^) with mean + SEM. RM one-way ANOVA (Šídák's multiple comparisons test) with P* < 0.05, P*** < 0.001. **C** Stimulation with immobilized VCAM-1/ICAM-1 induces mRNA expression of *Il1a, Il1b, Il6, Tnf* and *Cxcl10* in total bone marrow cells (BMC) of *Vav1-Cre x Jak2*^+*/*+^ (*n* = 3) and *Vav1-Cre x Jak2*^*VF/*+^ mice (*n* = 2) in comparison to BSA (grey columns). Data are shown as fold change (2^−ΔΔCT^) with mean + SEM. **D** Upon adhesion to VCAM-1/ICAM-1, the formation of β1-integrin clusters in 32D JAK2-WT (blue; *n* = 144 cells) and 32D JAK2-VF cells (red; *n* = 96 cells) is measured by fluorescence microscopy (Created in BioRender. Haage, R. (2025) https://BioRender.com/e52r401). The clusters per cell (upper middle) and surface area per cell in pixel (upper right) are increased in the 32D JAK2-VF cells after adhesion on immobilized VCAM-1/ICAM-1 (15 min). Data are shown as mean + SEM. Mann–Whitney test with P**** < 0.0001. Representative fluorescence microscopy images are shown (lower panel). The brightness of these images has been normalized to the highest value and is presented in false colors, ranging from dark blue (low fluorescence intensity) to bright yellow (high fluorescence intensity). The scale bar represents 10 µm

The murine myeloid progenitor 32D cell model expressing JAK2-V617F (JAK2-VF) versus JAK2 wild type (JAK2-WT) in combination with EPOR was employed and has been described previously [12, 13, 50]. This model has been widely used to characterize the JAK2-VF induced changes in signaling and growth-factor independent proliferation [12, 13, 50]. 32D JAK2-WT and 32D JAK2-VF cells were stimulated with soluble or immobilized adhesion molecules VCAM-1/ICAM-1 and then analyzed for cytokine mRNA expression. VCAM-1 and ICAM-1 are the ligands of the major leukocyte integrins VLA-4 and LFA-1, respectively. We focused on the cytokine mRNA species *Il1a, Il1b, Il6, Tnf,* and *Cxcl10*. These cytokines have been shown to play an important role in pathophysiology of JAK2-VF-positive MPNs [28–31, 51–58]. Adhesion to immobilized VCAM-1/ICAM-1 resulted in a significant increase in *Il1a* (147.4-fold) and *Il1b* (4.7-fold) mRNA expression (Fig. 1A, right upper panels). Moreover, the JAK2-VF mutation was found to significantly enhance VCAM-1/ICAM-1 mediated mRNA expression, displaying a 2941.8-fold and 214.8-fold increase, respectively (Fig. 1A, right upper panels). Interestingly, stimulation with soluble VCAM-1/ICAM-1 was not sufficient to induce a significant change in *Il1a* and *Il1b* mRNA expression (Fig. 1A, right upper panels). Stimulation with immobilized VCAM-1/ICAM-1 also resulted in significant induction of *Il6* and *Tnf* mRNAs (Fig. 1A, left lower and middle lower panels). However, *Il6* and *Tnf* mRNA expression was not further enhanced by JAK2-VF. *Cxcl10* mRNA expression was only slightly induced upon adhesion to immobilized ligand, with fold changes of 2.3 and 4.2, respectively (Fig. 1A, right lower panel). Interestingly, IL-1α further increased mRNA expression of inflammatory cytokines upon VCAM-1/ICAM-1 stimulation (Supplementary Fig. 1).

To investigate whether VCAM-1/ICAM-1 stimulation would also induce mRNA expression of inflammatory cytokines in primary hematopoietic cells, we utilized granulocytes and whole bone marrow (WBM) cells isolated from JAK2-VF knock-in mice [13, 47]. In this mouse model of MPN, expression of JAK2-VF is under the control of the endogenous murine Jak2 promoter [47]. Physiological expression of JAK2-VF causes a lethal MPN-like disease. Since in vitro culture of primary hematopoietic cells requires addition of growth-factors and cytokines which would interfere with the VCAM-1/ICAM-1 induced changes, granulocytes and WBM cells were stimulated with immobilized VCAM-1/ICAM-1 in FCS supplemented RPMI medium for 1 h, only. Granulocytes play an important role in the development and maintenance of MPN [59]. Therefore, this cell type was of particular interest for our investigation. Upon adhesion to immobilized VCAM-1/ICAM-1, mRNA expression levels were strongly increased 26.0-fold and 13.1-fold for *Il1a* and 6.8-fold and 7.4-fold for *Il1b* in *Jak2*^+*/*+^ and *Jak2*^*VF/*+^ granulocytes, respectively (Fig. 1B). Thus, here, the JAK2-VF mutation was not found to enhance VCAM-1/ICAM-1 mediated mRNA expression. Interestingly, in WBM cells, the increase of *Il1a, Il1b, Il6, Tnf* and *Cxcl10* mRNA expression upon adhesion to VCAM-1/ICAM-1, was less pronounced, but was clearly detectable (Fig. 1C). Additionally, lineage-negative cells including hematopoietic stem and progenitor cells, isolated monocytes and B cells were also examined and showed adhesion-induced mRNA cytokine expression of *Il1a* and *Il1b* (Supplementary Fig. 2). Thus, VCAM-1/ICAM-1 induced mRNA expression of inflammatory cytokines was detectable in various hematopoietic cell populations, although divergent expression patterns indicate cell type-specific regulatory mechanisms. Together, these data demonstrate that adhesion to VCAM-1/ICAM-1 coated surface fosters mRNA induction of inflammatory cytokines in myeloid 32D cells and in primary hematopoietic cells. In the 32D cell model, the JAK2-VF mutation served to further increase the mRNA levels of inflammatory cytokines. Another interesting aspect of these experiments was that ligand immobilization appears as a prerequisite for VCAM-1/ICAM-1 induced mRNA cytokine expression. This is in line with previously published data suggesting that ligand immobilization ensures that shear forces impact on integrin receptors upon ligand binding which then activate outside-in signaling [17].

Previous reports also indicate that integrin clustering and shear stress is necessary for the full activation of integrins and of integrin outside-in signaling [17, 60, 61]. To investigate integrin clustering in 32D cells, we employed fluorescence microscopy to examine the formation of CD29 (β1 integrin) clusters upon adhesion to immobilized VCAM-1 and ICAM-1. Upon binding to immobilized VCAM-1/ICAM-1, integrin β1 (CD29) clusters were readily detectable and the number of clusters per cell was significantly increased in 32D JAK2-VF cells (1.6-fold) as compared to JAK2-WT cells (Fig. 1D; upper middle panel). The area of the cell surface, which is in contact with the VCAM-1/ICAM-1 coated plate, was also significantly increased in the JAK2-VF-mutated cells (1.8-fold) in comparison to the JAK2-WT cells (Fig. 1D; upper right panel). Representative images of VCAM-1/ICAM-1 stimulated 32D JAK2-WT cells and of unstimulated (control) and VCAM-1/ICAM-1 stimulated 32D JAK2-VF cells are shown in Fig. 1D lower left, middle and right panel, respectively. The differences in β1 integrin clustering between 32D JAK2-WT and 32D JAK2-VF cells upon VCAM-1/ICAM-1 stimulation are shown in detail in Supplementary Fig. 3. Together, this data demonstrates that β1 integrin clustering induced upon stimulation with immobilized VCAM-1/ICAM-1 is enhanced by JAK2-VF.

### Outside-in signaling of β1 and β2 integrins is activated upon VCAM-1/ICAM-1 stimulation

**Fig. 2:**
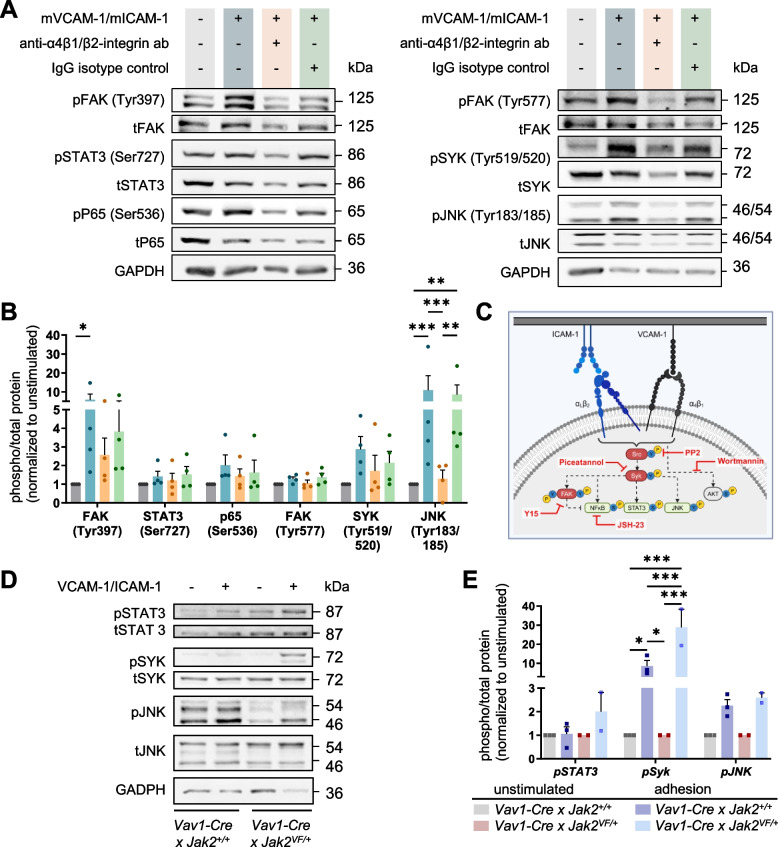
Outside-in signaling of β1/β2 integrins is activated upon adhesion to VCAM-1/ICAM-1. **A-B** Adhesion of 32D JAK2-VF cells on VCAM-1/ICAM-1 (15 min; blue; *n* = 4) induces phosphorylation of canonical integrin outside-in signaling in a VLA4/β2-integrin-specific manner. Pre-incubation with 5 µg/ml anti-VLA4/β2-integrin antibody (orange; *n* = 4) led to a reduction in adhesion-induced phosphorylation, while the corresponding IgG isotype control (5 µg/ml; green; *n* = 4) remained largely unaltered. **A** Representative Western blots of *n* = 4 replicates are shown. **B** Semi-quantification of phospho-signals derived from Western blots (*n* = 4) is depicted as phosphorylated (p) to total (t) protein signals normalized to the unstimulated control (BSA). 2-Way ANOVA P* < 0.05, P** < 0.01, P*** < 0.001 (**C**) Model of outside-in signaling upon adhesion to VCAM-1/ICAM-1. For characterization of outside-in signaling upon VCAM-1/ICAM-1 stimulation, a panel of representative signaling molecules (SRC, SYK, FAK, PI3K and NFκB) was investigated using the following inhibitors: PP2 (5 µM), Piceatannol (30 µM), Y15 (25 µM), Wortmannin (50 nM) and JSH-23 (50 µM), respectively. Results are given in Table 1. The proposed signaling model derived from this data is depicted as a cartoon (Created in BioRender. Haage, R. (2025) https://BioRender.com/q22h778). **D-E** VCAM-1/ICAM-1 stimulation (15 min) using total bone marrow cells of *Vav1-Cre x Jak2*^+*/*+^ and *Vav1-Cre x Jak2*.^*VF/*+^ mice induced the phosphorylation of STAT3 (Ser727), SYK (Tyr519/520) and JNK (Tyr183/185) in comparison to unstimulated cells (BSA). **D** Representative Western blots (*n* = 2–3) and (**E**) semi-quantification (*n* = 2–3) are shown. Data are shown as mean + SEM. 2-Way ANOVA P* < 0.05, P** < 0.01, P*** < 0.001

Previously, we reported that expression of JAK2-VF results in the constitutive activation of **in**side-out signaling of β1 and β2 integrins, which translates into increased adhesion of mouse and human granulocytes on VCAM and ICAM-1 [12, 13]. To investigate the impact of VCAM-1/ICAM-1 stimulation on **out**side-in signaling we utilized 32D JAK2-VF cells. Interestingly, adhesion to VCAM-1/ICAM-1 strongly up-regulated the phosphorylation of FAK at Tyr397 and to a lesser extent at Tyr 577 (Fig. 2A-B). It has been reported that activation of FAK by integrin clustering leads to auto-phosphorylation at Tyr397, which is a binding site for the Src family kinases and for PI3K and PLCγ [62–65]. Recruitment of Src family kinases results in the phosphorylation of Tyr407, Tyr576, and Tyr577 in the catalytic domain of FAK [66, 67]. STAT3 at site Ser727, NFκB (p65) at site Ser536 and SYK at site Tyr519/520 also showed modestly increased phosphorylation levels upon VCAM-1/ICAM-1 stimulation (Fig. 2A-B), although this was not statistically significant. Phosphorylation at Ser727 of STAT3 through MAPK and mTOR pathways was reported to regulate transcriptional activation of STAT3 [68, 69]. Notably, a strong increase in the phosphorylation of JNK at site Tyr183/185 of was observed (Fig. 2A-B). VCAM-1/ICAM-1 induced phosphorylation levels were reduced by pre-incubation with anti-integrin antibody by 14.9% to 88.2%, whereas pre-incubation with the corresponding IgG control did not result in major changes (Fig. 2A-B). In conclusion, our data indicate that adhesion to immobilized VCAM-1/ICAM-1 induces canonical outside-in signaling in JAK2-VF-positive 32D cells in a VLA4/β2-integrin-specific manner.

To gain further insight into the outside-in signaling process, we investigated the inhibitory action of a panel of signaling molecule inhibitors, including PP2, Y15, Piceatannol (Pic), Wortmannin (Wort), and JSH-23, on Src, FAK, SYK, PI3K, and NFκB, respectively. The results are presented as a summary in Table 1. This data suggested that inhibition of SRC and SYK negatively affects phosphorylation levels of all signaling nodes investigated. Thus, SRC and SYK may be placed upstream of the majority of the analyzed signaling molecules, while PI3K activation appeared to be less important for most of the analyzed outside-in signaling molecules. This result is depicted in Fig. 1C which proposes a model of outside-in signaling upon adhesion to VCAM-1/ICAM-1 in 32D JAK2-VF cells.

**Table 1:** Characterization of signaling nodes involved in outside-in signaling of 32D JAK2-VF/EPOR cells upon VCAM-1/ICAM-1 stimulation

	PP2	Piceatannol	Y15	Wortmannin	JSH-23
Signaling node investigated	Src inhibitor	Syk inhibitor	FAK inhibitor	PI3K inhibitor	NFκB inhibitor
FAK (Tyr577)	−17	−28	−38	21	n.d
FAK (Tyr397)	−100	−14	−43	−25	n.d
STAT3 (Ser727)	−23	−50	5	−26	−37*
SYK (Tyr519/520)	−60	−50	−2	−19	−37
p65 (Ser536)	−63	−24	239	10	−31*
AKT (Ser473)	n.d	n.d	n.d	−100	n.d
JNK (Tyr183/185)	−62	−75	1	n.d	−54

Various signaling molecule inhibitors were tested for their action on major signaling nodes of integrin outside-in signaling. Shown is the percentage change (%) in the phosphorylated/total protein signal of signaling molecules indicated as evaluated from Western blots (each 3 replicates; data not shown) using ImageJ software

The phosphorylated/total protein signals obtained from inhibitor treated cells upon adhesion to immobilized VCAM-1/ICAM-1 were compared to the phosphorylated/total protein signals of untreated 32D JAK2-VF/EPOR cells upon adhesion to VCAM-1/ICAM-1 (control). Each 3 replicates were generated and the change of mean (out of 3 replicates) phosphorylation levels is given in %. A negative value indicates down-regulation of phosphorylation, a positive value indicates up-regulation of phosphorylation upon inhibitor treatment during adhesion. SRC, SYK, FAK, PI3K and NFκB were inhibited by PP2 (5 µM), Piceatannol (30 µM), Y15 (25 µM), Wortmannin (50 nM) and JSH-23 (50 µM), respectively. * measured in the nucleus. n.d. – not detected

The inhibitor concentrations were evaluated in pilot experiments using three different concentrations of each inhibitor (PP2: 1/5/10 µM; Piceatannol: 30/60/90 µM; Y15: 5/25/50 µM; Wortmannin: 10/50/100 nM; JSH-23: 10/50/100 µM). The respective inhibitor concentration chosen was shown to inhibit the phosphorylation state of the respective target protein in unstimulated cells. An exception is Wortmannin; here, the lowest concentration that completely inhibited AKT was chosen

To investigate outside-in signaling in primary hematopoietic cells, WBM cells of *Jak2*^+*/*+^ and *Jak2*^*VF/*+^ mice were utilized. A 8.5- and 2.3-fold increase in the phosphorylation of SYK and JNK was observed in *Jak2*^+*/*+^ cells upon adhesion to immobilized VCAM-1/ICAM-1 (Fig. 2D-E). Phosphorylation of two adjacent tyrosines in the activation loop of SYK kinase domain, Tyr519/Tyr520, which is equivalent to phosphorylation at Tyr525/526 of human SYK has been reported to be essential for SYK function [70]. Remarkably, the adhesion-mediated phosphorylation of SYK was further increased by JAK2-VF. These results align with the 32D cell results and demonstrate that canonical outside-in signaling of β1/β2 integrins is detected in primary hematopoietic cells.

### Signaling nodes involved in VCAM-1/ICAM-1-stimulated expression of *Il1a* and *Il1b* mRNAs

**Fig. 3:**
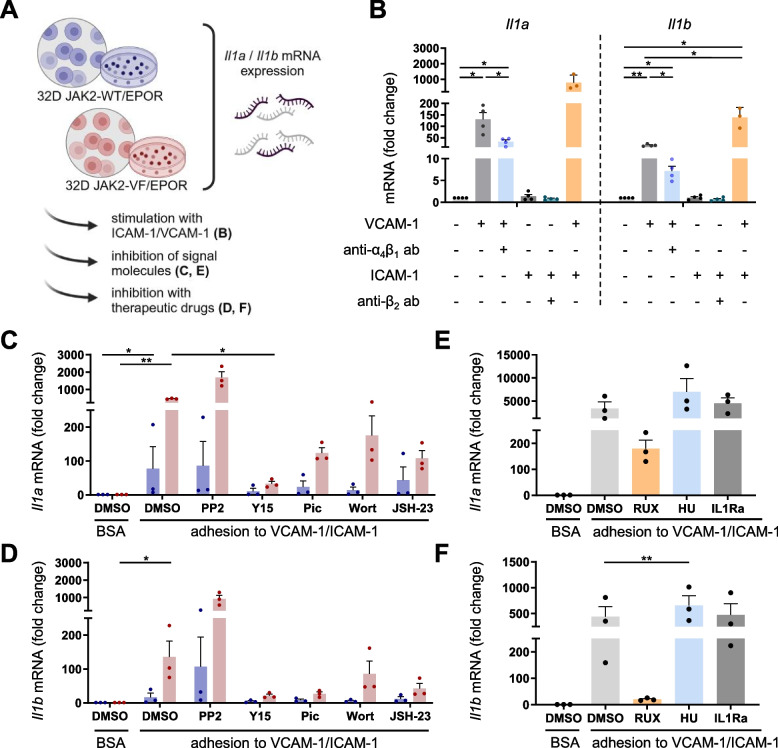
Characterization of integrin outside-in signaling nodes involved in VCAM-1/ICAM-1-induced mRNA expression of IL-1α and IL-1β. **A** Cartoon depicting the experimental design (Created in BioRender. Haage, R. (2025) https://BioRender.com/w21u020). **B** In 32D JAK2-VF cells, *Il1a* and *Il1b* mRNA expression is induced by the VLA4-VCAM-1 interaction (*n* = 4) but not by the β2-integrin-ICAM-1 interaction (*n* = 4). Specificity was tested by pre-incubation using 5 µg/ml anti-VLA4 or anti-β2-integrin antibody (*n* = 4). The stimulation of VCAM-1 and ICAM-1 in combination resulted in a further increase in adhesion-induced mRNA expression (orange; *n* = 3). **C-D** 32D JAK2-WT (blue) and 32D JAK2-VF (red) cells were stimulated with VCAM-1/ICAM-1 and various inhibitors were applied. The expression levels of (**C**) *Il1a* (*n* = 3) and **(D)** *Il1b* mRNA (*n* = 3) were measured upon inhibition of signaling molecules, including SRC (5 µM PP2), FAK (25 µM Y15), SYK (30 µM Piceatannol (Pic)), PI3K (50 nM Wortmannin (Wort)) and NFκB (50 µM JSH-23). **E–F** Clinical therapeutic drugs such as Ruxolitinib (2 µM RUX; orange) and Hydroxyurea (0.5 mM HU; blue) were tested for their effects on adhesion-induced (**E**) *Il1a* and (**F**) *Il1b* mRNA expression. To exclude the possibility of autocrine IL-1 signaling, IL1Ra was applied (dark grey). mRNA expression was measured by qPCR upon 3 h of stimulation and the resulting data are shown as fold change (2^−ΔΔCT^) as mean + SEM (*n* = 3–4). **B** Mixed-effects analysis (uncorrected Fisher´s LSD), **C-D** Friedman test (uncorrected Dunn´s test), **E–F** RM one-way ANOVA (uncorrected Fisher´s LSD) with P* < 0.05, P** < 0.01

VCAM-1 and ICAM-1 are natural ligands of VLA4 (α_4_β_1_) and LFA1 (α_L_β_2_) or Mac1 (α_M_β_2_) integrins, respectively. Both integrin classes (β1/β2-integrins) are expressed on 32D JAK2-WT and JAK2-VF cells (Gupta et al.[12]; Gupta and Fischer, unpublished data). First, we aimed to investigate the role of the ligand specificity in 32D JAK2-VF cells. Thus, VCAM-1-VLA4 and the ICAM-1-β2-integrin interactions were analyzed separately (Fig. 3A-B). The VCAM-1-VLA4 interaction induced a significant increase in *Il1a* (130.7-fold) and *Il1b* (17.8-fold) mRNA expression (Fig. 3B). The specificity of VLA4 was controlled by pre- and co-incubation with an anti-VLA4-antibody. This revealed a significant inhibition of the adhesion-induced *Il1a* (−74.6%) and *Il1b* (−59.7%) mRNA levels (Fig. 3B). Notably, adhesion to ICAM-1 alone was not sufficient to induce mRNA expression. However, interestingly, the addition of ICAM-1 had an amplifying effect on the VCAM-1-induced mRNA expression of *Il1a* (6.0-fold) and *Il1b* (7.8-fold).

Given that both VCAM-1 and ICAM-1 are expressed on endothelial cells and on other matrices [19], the subsequent experiments employed combined VCAM-1/ICAM-1 stimulation. In order to gain a more detailed understanding of the signaling molecules involved in adhesion-induced mRNA expression of *Il1a and Il1b*, a panel of inhibitors was investigated. The same inhibitors (PP2, Y15, Pic, Wort, JSH-23) were used as for the characterization of integrin outside-in signaling depicted in Table 1. As shown in Fig. 1, VCAM-1/ICAM-1 stimulation strongly increased *Il1a* and *Il1b* mRNA levels in JAK2-WT and JAK2-VF cells, whereby presence of JAK2-VF led to a further increase (Fig. 3C-D). Unexpectedly, PP2 (Src-inhibitor) did not downregulate the adhesion-induced mRNA expression of *Il1a* and *Il1b* (Fig. 3C-D)*.* Y15, Piceatannol, Wortmannin and JSH-23 reduced the VCAM-1/ICAM-1-induced *Il1a* and *Il1b* mRNA expression to a level between −37% and −93% in JAK2-WT and JAK2-VF cells (Fig. 3C-D). Y15 resulted in near-complete inhibition of adhesion-induced *Il1a* expression in both cell lines, with a reduction of −86% and −93%, respectively. The *Il1b* mRNA level was reduced by −75% and −85%, respectively (Fig. 3C-D). These results demonstrate that Y15 is the most potent inhibitor in this analysis. This suggests a crucial role of FAK in the adhesion-induced cytokine mRNA expression. However, the data also indicates the involvement of multiple signaling pathways in VCAM-1/ICAM-1-induced mRNA expression of *Il1a* and *Il1b* (Fig. 3C-D).

Clinically important drugs in MPN treatment are the JAK1/2 inhibitor Ruxolitinib (Rux) and the DNA synthesis inhibitor hydroxyurea (HU). Therefore, their actions in adhesion-mediated mRNA induction were also examined. Strikingly, the JAK1/2 inhibitor Ruxolitinib strongly reduced adhesion-induced *Il1a* (−94.8%) and *Il1b* (−95.5%) mRNA in 32D JAK2-VF cells (Fig. 3E-F). Interestingly, HU led to a further increase in the levels of *Il1a* (2.0-fold) and *Il1b (1.5-fold)* mRNA. Furthermore, the impact of an IL1-receptor antagonist (IL1Ra) was investigated, since IL1Ra is discussed as a potential new treatment in MPN [28]. Here, no significant changes were observed (Fig. 3E-F). Thus, these results indicate a JAK-dependent and DNA synthesis-independent adhesion-induced mRNA expression of *Il1a* and *Il1b*. The contribution of any potentially existing IL-1 autocrine or paracrine actions could be ruled out by the employment of IL1Ra.

### VCAM-1/ICAM-1 stimulation up-regulates IL-1α and IL-1β protein levels in 32D cells but not in primary granulocytes

**Fig. 4:**
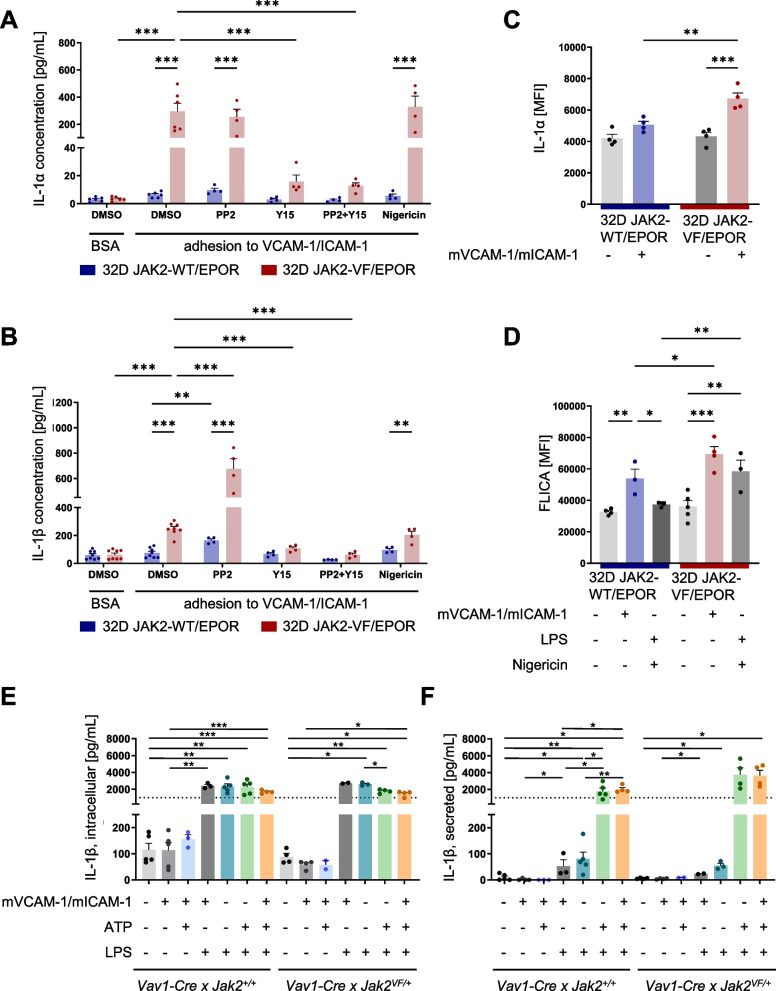
Stimulation by VCAM-1/ICAM-1 induces protein expression of IL-1α/β in 32D JAK2-WT and 32D JAK2-VF cells. **A-B** VCAM-1/ICAM-1 stimulation increases intracellular (**A**) IL-1α and (**B**) IL-1β protein levels in 32D JAK2-WT (blue) and 32D JAK2-VF cells (red) in comparison to the BSA control (*n* = 4–6). After 6 h of stimulation, intracellular, mature IL-1α and IL-1β was measured by ELISA. The effects of PP2 (5 µM), Y15 (25 µM) and nigericin (5 µM) were investigated. 2-Way ANOVA with P* < 0.05, P** < 0.01, P*** < 0.001. **C** Following 24 h VCAM-1/ICAM-1 stimulation, significantly increased IL-1α surface expression was detected by flow cytometry in 32D JAK2-VF (red; *n* = 4) cells but not in 32D JAK2-WT cells (blue; *n* = 4). RM one-way ANOVA (Fisher´s LSD) with P** < 0.01, P*** < 0.001. **D** Upon VCAM-1/ICAM-1 stimulation (6 h), the presence of active caspase 1 was measured by FLICA (FAM-YVAD-FMK) using flow cytometry in 32D JAK2-WT cells (blue; *n* = 3) and 32D JAK2-VF (red; *n* = 4). As a positive control, cells were stimulated with LPS (100 ng/ml) and nigericin (5 µM). Ordinary one-way ANOVA with P* < 0.05, P** < 0.01, P*** < 0.001. **E** Intracellular and (**F**) secreted IL-1β protein levels of granulocytes isolated from *Vav1-Cre x Jak2*^+*/*+^ and *Vav1-Cre x Jak2*.^*VF/*+^ mice upon 6 h VCAM-1/ICAM-1 stimulation (*n* = 5) were measured. LPS (100 ng/ml) and ATP (3 mM) were utilized to investigate the effects of the inflammasome cascade on VCAM-1/ICAM-1 stimulation of IL-1α and IL-1β. They also served as positive controls. Data are shown as mean + SEM. Mixed-effects analysis (uncorrected Fisher´s LSD) with P* < 0.05, P** < 0.01, P*** < 0.001

Next, we examined whether VCAM-1/ICAM-1 stimulation results in an increase in inflammatory cytokines at the protein level. No differences in intracellular IL-1α and IL-1β concentrations were observed between unstimulated 32D JAK2-WT and 32D JAK2-VF cells (Fig. 4A-B). However, in line with elevated mRNA levels shown above, a significant increase in intracellular IL-1α (40.0-fold) and IL-1β (3.1-fold) concentrations was observed upon VCAM-1/ICAM-1 stimulation in JAK2-VF cells. In accordance with the data obtained from the mRNA analysis, Y15 significantly reduced VCAM-1/ICAM-1-induced intracellular IL-1α and IL-1β levels in JAK2-VF cells, while PP2 again showed no inhibitory effects (Fig. 4A-B). The combination of PP2 and Y15 neutralized the amplifying impact of PP2 on intracellular IL-1 concentrations to a level comparable to that achieved with Y15 alone (Fig. 4A-B). Nigericin (NLRP3 activator) is a K-ionophore, which is described to induce IL-1 secretion [49]. Co-stimulation of VCAM-1/ICAM-1 treated cells with Nigericin to activate the inflammasome revealed no significant differences in intracellular IL-1 concentrations as compared to the DMSO control across both cell lines.

Pro- and mature IL-1α is biological active [37] and is localized in different sites thereby displaying different modes of action [71–74]. IL-1α is capable of being expressed on the cell surface [72, 75–77], thereby inducing a local immune response [74]. Therefore, the IL-1α surface expression was examined after 24 h adhesion to VCAM-1/ICAM-1. Upon stimulation with immobilized VCAM-1 and ICAM-1, the IL-1α surface expression was slightly increased (20.1%) in JAK2-WT cells and strongly increased (55.3%) in JAK2-VF cells (Fig. 4C). Notably, the presence of JAK2-VF significantly enhanced adhesion-induced IL-1α surface expression (32.9%) compared to JAK2-WT cells, while no differences were found in the BSA-stimulated controls.

To gain further insight into IL-1 processing, a FLICA assay was used to monitor active caspase 1 and pyroptosis upon integrin stimulation. VCAM-1/ICAM-1, significantly enhanced the FLICA signal (shown as mean fluorescence intensity—MFI) in JAK2-WT (3.4-fold) and in JAK2-VF (4.8-fold) cells, respectively (Fig. 4D). Interestingly, VCAM-1/ICAM-1 stimulation resulted in a higher FLICA signal as the positive control (LPS/Nigericin) and was further increased by JAK2-VF. The percentage of active caspase 1-positive alive cells as well as pyroptotic cells was also increased upon VCAM-1/ICAM-1 stimulation in 32D JAK2-WT and JAK2-VF cells (Supplementary Fig. 4). In summary, adhesion to VCAM-1/ICAM-1 induced intracellular and membrane bound IL-1α expression. It also increased active caspase 1 necessary for processing pro-IL-1β and thus resulted in an up-regulation of intracellular mature IL-1β. Notably, the observed changes were further amplified by the presence of JAK2-VF.

Next, we examined the intracellular IL-1α and IL-1β protein concentrations and secretion of IL-1β in primary granulocytes isolated from *Jak2*^+*/*+^ and *Jak2*^*VF/*+^ mice upon 6 h of VCAM-1/ICAM-1 stimulation (Fig. 4E-F and data not shown). Surprisingly, in vitro adhesion of isolated granulocytes on immobilized VCAM-1/ICAM-1 was not sufficient to increase intracellular IL-1β or to induce IL-1β secretion, either alone or in combination with LPS or ATP (Fig. 4E-F). Therefore, we conclude that adhesion to VCAM-1/ICAM-1 does not function as a priming or activation signal for IL-1β maturation in vitro. Intracellular IL-1α was also not detectable, neither in unstimulated cells nor in VCAM-1/ICAM-1 stimulated cells (data not shown). However, in view of the positive control signal, the presence of general defects in IL-1 maturation and secretion in the cells can be excluded.

### Adhesion to VCAM-1/ICAM-1 induces an inflammatory cytokine mRNA signature in primary granulocytes

**Fig. 5:**
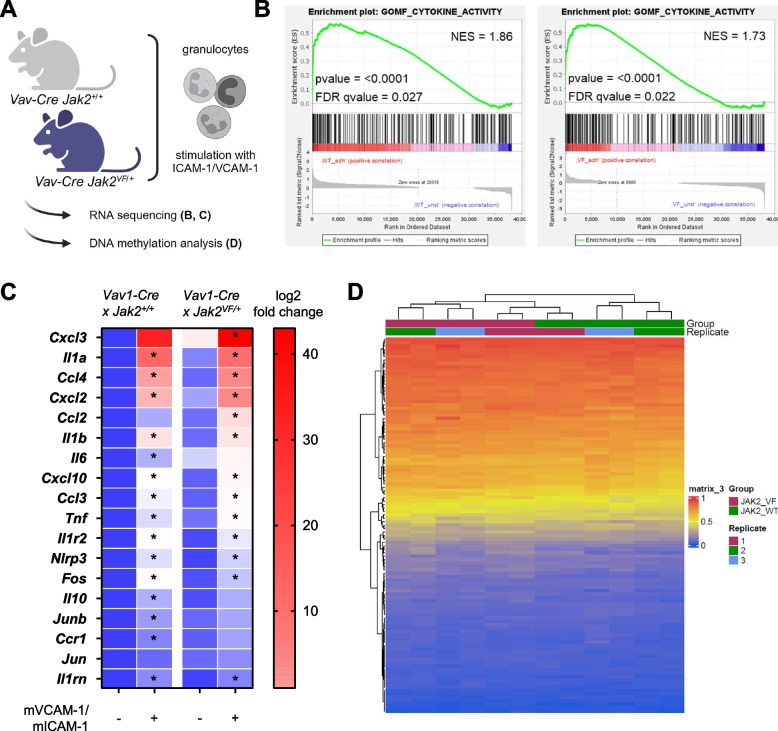
RNASeq in primary granulocytes shows a VCAM-1/ICAM-1 activated inflammatory cytokine signature. **A** Granulocytes isolated from *Vav1-Cre x Jak2*^+*/*+^ and *Vav1-Cre x Jak2*^*VF/*+^ mice were stimulated for 1 h using immobilized VCAM-1/ICAM-1 or BSA (control). RNA sequencing (*n* = 4) and DNA-methylation analysis (*n* = 3) were performed (Created in BioRender. Haage, R. (2025) https://BioRender.com/n22t547). **B** Gene set enrichment analysis (GSEA) profiles of cytokine activity in *Jak2-WT* granulocytes (left) and in *Jak2-V617F* mutated granulocytes (right) upon adhesion to VCAM-1/ICAM-1 vs. unstimulated control (BSA) are shown. The normalized enrichment score (NES), the *p*-value and the false discovery rate (FDR) are given. **C** Changes in mRNA expression (log2 fold change) of 18 representative inflammatory genes of the GSEA gene set are depicted and shown as heatmap normalized to unstimulated (BSA stimulated) *Jak2-WT* granulocytes. The scale ranges from blue (representing 1) to bright red, indicating the highest observed increase in log2 fold change. Unstimulated (BSA) and VCAM-1/ICAM-1 stimulated granulocytes of *Vav1-Cre x Jak2*^+*/*+^ and *Vav1-Cre x Jak2*^*VF/*+^ mice were compared. Differentially expressed genes (deseq2, log2 fold > 1, padj < 0.05) are indicated with an asterisk *. The unstimulated (BSA) controls have been partly depicted in a previous publication [78]. Please note that in Fig. 5 C the log2 fold change covers a range from 0 up to > 40. **D** A DNA-methylation array was performed upon VCAM-1/ICAM-1 stimulation (1 h) of isolated granulocytes from *Vav1-Cre x Jak2*^+*/*+^ (JAK2_WT; *n* = 3) and *Vav1-Cre x Jak2*.^*VF/*+^ (JAK2_VF; *n* = 3) mice. The CpG methylation islands of the genes shown in (C) were depicted in a heatmap (blue represents 0—not methylated, red represents 1 – fully methylated)

Granulocytes are important mediators of inflammation in MPN [78]. Therefore, we sought to investigate the effects of adhesion to VCAM-1/ICAM-1 on induction of inflammatory mRNA species in a broader range employing RNA sequencing (RNAseq) on ex vivo VCAM-1/ICAM-1 stimulated granulocytes from mice (Fig. 5A). Principal component analysis (PCA), revealed a distinct separation between *Jak2*^+*/*+^ and *Jak2*^*VF/*+^ granulocytes as well as between VCAM-1/ICAM-1 stimulated and unstimulated (BSA) cells (Supplementary Fig. 5). Gene set enrichment analysis (GSEA) identified a large number of enriched gene sets. Among the top 5 enriched genes set, we found “GOMF_CYTOKINE_ACTIVITY” (GO:0005125), which comprises a large number of inflammatory markers (Fig. 5B). The results demonstrated a comparable enrichment of positively regulated genes upon VCAM-1/ICAM-1 stimulation both in *Jak2*^+*/*+^ and *Jak2*^*VF/*+^ granulocytes, with a normalized enrichment score (NES) of 1.86 and 1.73, and a false discovery rate (FDR) of 0.027 and 0.022, respectively (Fig. 5B). The fold change of 18 representative inflammatory markers out of 236 “GOMF_CYTOKINE_ACTIVITY” genes are shown in Fig. 5C, normalized to unstimulated *Jak2*^+*/*+^ granulocytes (log2fold change of TPM). We observed a massive up-regulation of genes encoding inflammatory cytokines (e.g. *Cxcl3, IL1a*, *Ccl4, IL1b, IL6, Tnf*), transcription factors and other inflammatory factors (e.g. *Nlrp3*) upon VCAM-1/ICAM-1 stimulation in both genotypes (Fig. 5C and data not shown).

In addition to driver mutations as JAK2-VF, mutations in genes regulating epigenetic processes have also been identified in MPN patients [79–81]. To assess the role of epigenetic factors in the observed differences between unstimulated granulocytes isolated from *Jak2*^+*/*+^ and *Jak2*^*VF/*+^ mice, and between unstimulated and VCAM-1/ICAM-1 stimulated granulocytes, DNA methylation was analyzed (Fig. 5D). The heatmap depicted in Fig. 5D represents the collective methylation status of all CpGs of the genes illustrated in Fig. 5C. No apparent differences in DNA methylation between unstimulated and VCAM-1/ICAM-1 stimulated granulocytes were detectable; consequently, they were grouped together in the heatmap. The presented results collectively indicate that there is no evidence of an epigenetic change induced by VCAM-1/ICAM-1 stimulation in vitro.

### In vivo relation between integrin activation and serum protein levels of IL-1α

**Fig. 6:**
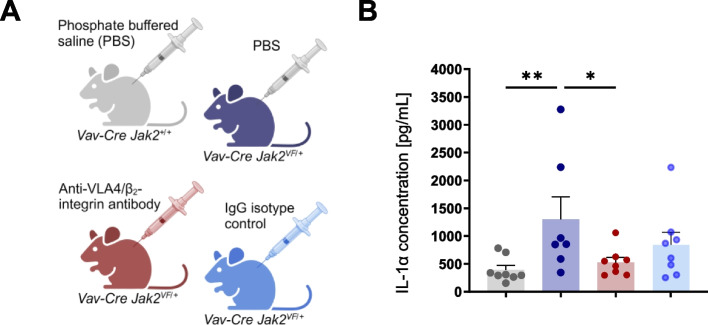
Inhibition of VCAM-1/ICAM-1 mediated integrin signaling in vivo down-regulates IL-1α serum levels of JAK2^VF/+^ mice. **A** 10 weeks old *Vav1-Cre x Jak2*^+*/*+^ (grey; *n* = 8) and *Vav1-Cre x Jak2*^*V*F/+^ mice (dark blue; *n* = 7) were injected with a single intraperitoneal dose of PBS (200 µl) to control for injection-dependent artefacts. Results were compared with V*av1-Cre x Jak2*.^*VF/*+^ mice that had received a single dose (200 µl) of anti-VLA4/β2-integrin antibody (red; 200 µg, *n* = 8) or the corresponding IgG control (light blue; 200 µg, *n* = 8) (Created in BioRender. Haage, R. (2025) https://BioRender.com/v80w629). On day 8 upon injections, serum was collected and cytokine serum analysis was performed. **B** IL-1α serum concentrations are shown as mean + SEM. Ordinary One-Way ANOVA P* < 0.05, P** < 0.01

In the present paper, we showed activated outside-in signaling, inflammatory cytokine mRNA expression, caspase 1 activation and elevated intracellular IL-1 concentrations upon VCAM-1/ICAM-1 stimulation. The additional effect of JAK2-VF varied between the results obtained in 32D JAK2 cells and in those from primary hematopoietic cells. Furthermore, the absence of detectable intracellular IL-1α and IL-1β proteins upon VCAM-1/ICAM-1 stimulation in primary granulocytes indicates that the necessary signals are missing under in vitro conditions. Therefore, we sought to investigate the impact of integrin activation in vivo on IL-1 serum concentrations in *Jak2*^*VF/*+^ mice (Fig. 6A). For IL-1β, the serum concentrations in the Vav-Cre JAK2-VF mouse model were below the detection limit of the assay. However, the concentration of IL-1α was significantly elevated in *Jak2*^*VF/*+^ mice (mean 1302 pg/ml) as compared to *Jak2*^+*/*+^ mice (mean 392 pg/ml) (Fig. 6B). To suppress the interaction between the integrin receptors VLA-4 and the β2 chain integrin with their ligands (mainly VCAM-1 and ICAM-1), neutralizing anti-integrin antibodies were employed in comparison to isotype IgG controls. Remarkably, upon administration of a single dose of the anti-α_4_β_1_/β_2_-integrin antibodies to *Jak2*^*VF/*+^ mice, the IL-1α serum concentration was significantly reduced by 59%. This indicates that inhibition of the interaction between β1/β2-integrin and VCAM-1/ICAM-1 in vivo is sufficient to reduce JAK2-VF-mediated elevated IL-1α concentrations in the serum of *Vav1-Cre x Jak2*^*VF/*+^ mice. In light of the data presented above, it is likely that up-regulation of IL-1α mRNA and protein levels in hematopoietic cells upon interaction of integrins with VCAM-1 and ICAM-1 is a contributing factor of elevated IL-1α serum levels in *Jak2*^*VF/*+^ mice. Interestingly, JAK2-VF mutated inflammatory leukocytes isolated from *Jak2*^*VF/*+^ mice (granulocytes, monocytes and macrophages) are attracted by IL-1α and showed increased migration towards an IL-1α gradient in vitro (Supplementary Fig. 6). This suggests that the presence of IL-1α in any tissue (e.g. peripheral blood or bone marrow) may also foster migration of granulocytes, monocytes and macrophages towards this tissue. Since these cells carry high levels of inflammatory cytokines, this mechanism may also contribute to up-regulation of inflammatory cytokines in the particular microenvironment.

## Discussion

The present study investigated the hypothesis that interaction of the major leukocyte β1 and β2 integrins with the adhesion molecules VCAM-1 and ICAM-1 induces inflammatory cytokines in hematopoietic cells. The focus of our study was to examine (i) cluster formation and outside-in signaling of integrins, (ii) induction of inflammatory cytokines at mRNA and protein levels and (iii) caspase 1 dependent and non-dependent IL-1 processing. As critical factors in the formation of integrin clusters, presence of active integrins, immobilized ligands with high ligand density, PIP2, and talin have been identified before [82]. Furthermore, integrin clustering has been demonstrated to induce outside-in signaling (e.g. activation of FAK[83] and SYK[84]) and to enhance avidity of integrins [85]. Interestingly, ligand immobilization of VCAM-1/ICAM-1 was required for the induction of integrin-mediated pro-inflammatory cytokine mRNA expression in our study. Since immobilized ligands foster integrin clustering, this suggests that formation of integrin clusters plays an important role in cytokine mRNA induction. The presence of the JAK2-V617F mutation enhanced the formation of β1-integrin clusters upon adhesion, and further increased mRNA expression levels of IL-1α and IL-1β in 32D myeloid progenitor cells. This is in line with the previously described JAK2-VF induced pre-activation of β1 and β2 integrins which results in a shift from the bent, low affinity conformation to the open, high affinity state [12, 13].

Upon adhesion of 32D JAK2-VF cells to VCAM-1/ICAM-1, a variety of outside-in signaling nodes were identified, including FAK, STAT3, SYK, NFκB, JNK and AKT. Our data shows that the VCAM-1-VLA4 interaction plays a dominant role in adhesion-induced mRNA expression of inflammatory cytokines. These findings are consistent with the observations of Yurochko et al. [86] and Zohlnhöfer et al. [87] described in monocytes. In contrast, other cytokine mRNAs (e.g., *Il8* [88]*, Ccl3* [89]) have been shown to be primarily expressed upon ICAM-1 stimulation. This suggests a mechanism that is both cytokine- and cell-type-specific. Interestingly, our study shows that the ICAM-1-β2 interaction has an additive effect on the VCAM-1-VLA4-induced mRNA expression of IL-1β. Further, the inhibitor experiments of signaling nodes clearly corroborated the pivotal role of FAK in the adhesion-induced mRNA expression of IL-1α. In addition, SYK, NFκB and AKT appear to also participate. NFκB, as a transcription factor, induces the transcription of pro-IL-1α [90] and pro-IL-1β [91]. AP-1 is another transcription factor of pro-IL-1α [92] and pro-IL-1β [93], which is activated by MAPK (p38, ERK, JNK) [94]. It has been demonstrated that AKT can activate the AP-1 transcription factor through the action of ERK and JNK [95]. Given that SYK is located upstream of both NFκB and JNK, it may stimulate NFκB- and/or AP-1-induced transcription of pro-IL-1α and pro-IL-1β. In contrast, SYK^−/−^ macrophages showed increased LPS-induced *Il1b* gene expression, suggesting a negative effect of SYK on the *Il1b* expression in macrophages [96]*.* Thus, it appears that SYK acts in a cell type specific manner in the regulation of *Il1b* mRNA induction.

Y15 (FAK-inhibitor) significantly reduced adhesion-induced mRNA expression of IL-1α at the highest level, strongly suggesting that FAK plays an essential role in outside-in signaling of integrins. This finding is consistent with previous reports that identified FAK as a critical initiating factor in diverse signaling pathways, including the activation pathways of MAPK (e.g., JNK [97]). The present data offer no evidence of FAK-related JNK activation, a finding that aligns with the observations reported by Snider et al. [98]. However, other signaling molecules, such as Rac1, may be involved [99]. Furthermore, FAK serves as a mediator between integrin and growth factor signaling, thereby amplifying integrin signaling [100]. It has been demonstrated that FAK can phosphorylate Src kinases, thereby achieving the maximum level of kinase activity [101, 102]. This indicates that the inhibition of FAK may impede the activity of various signaling pathways, thereby preventing the reinforcement of certain signaling nodes and ultimately reducing *Il1* expression. Of note, we are not aware that off-targets have been described when utilizing the FAK inhibitor Y15 at the concentration used in our study.

In 32D JAK2-VF cells, adhesion to VCAM-1/ICAM-1 resulted in a significant increase in IL-1α and IL-1β at the protein level. This supports our hypothesis that activation of β1/β2 integrins contributes to elevated levels of inflammatory cytokines in JAK2-VF positive cells. However, surprisingly, in primary hematopoietic cells isolated from JAK2-VF knock-in mice and their wild-type controls no changes in intracellular or secreted protein levels of IL-1α and IL-1β were noted upon adhesion to VCAM-1/ICAM-1. Nevertheless, our in vivo experimentation demonstrated that blocking the interaction of integrins with VCAM-1/ICAM-1 using neutralizing antibodies against VLA4/β2 integrins effectively downregulated the elevated IL-1α blood levels in JAK2-VF knock-in mice. We speculate that increased IL-1α blood levels result from the interaction of JAK2-VF and/or JAK2-WT hematopoietic cells with VCAM-1/ICAM-1 expressed on a variety of cells in vivo. Thus, the failure to detect VCAM-1/ICAM-1 induced protein synthesis of IL-1 in primary hematopoietic cells in vitro may indicate the need for a second signal, which appears to be present in vivo but not in vitro. It is known that IL-1 protein synthesis is not exclusively regulated by induction of mRNA but requires additional signals due to the strong inflammatory actions and pleiotropic effects of IL-1α released from hematopoietic cells.

The observed differences between the results obtained in the 32D JAK2/EPOR cells and those derived from the isolated primary cell populations may be attributable to the overexpression of JAK2-WT/JAK2-V617F in the 32D cells or due to immortalization of the cell line. This is a clear limitation of our study. Nevertheless, the 32D JAK2/EPOR cell line was useful to characterize basic principles in integrin outside-in signaling involved in mRNA induction of inflammatory cytokine. An example is identification of the involvement of Syk and JNK phosphorylation which was found both in 32D cells and in primary cells.

A difference in the differentiation state may also play a role to explain the differences between the results obtained in the 32D JAK2/EPOR cells and those derived from the isolated primary granulocytes and whole bone marrow cells. Interestingly, in undifferentiated lineage-negative cells but not in granulocytes or monocytes, a trend towards elevated Il1b, Il6 and Tnf mRNA levels in JAK2-V617F mutant cells compared to their wild-type counterparts was observed (Supplementary Fig. 2). This is similar to the situation observed in undifferentiated myeloid progenitor-like 32D cells. Further research in primary hematopoietic cells isolated from JAK2-V617F mice and MPN patients is necessary to address these issues.

As another novel aspect, we showed an increase in IL-1α surface expression following VCAM-1/ICAM-1 stimulation in 32D JAK2-VF cells. Previously, the pro-inflammatory nature of surface IL-1α expression was demonstrated, including the capacity to induce a local immune response and to recruit additional immune cells [45, 103, 104]. This indicates potential for the induction of a pathogenic local immune response in JAK2-VF-positive MPN. Along this line, the existence of autoimmune pathology has been described in some MPN patients [105].

**Fig. 7:**
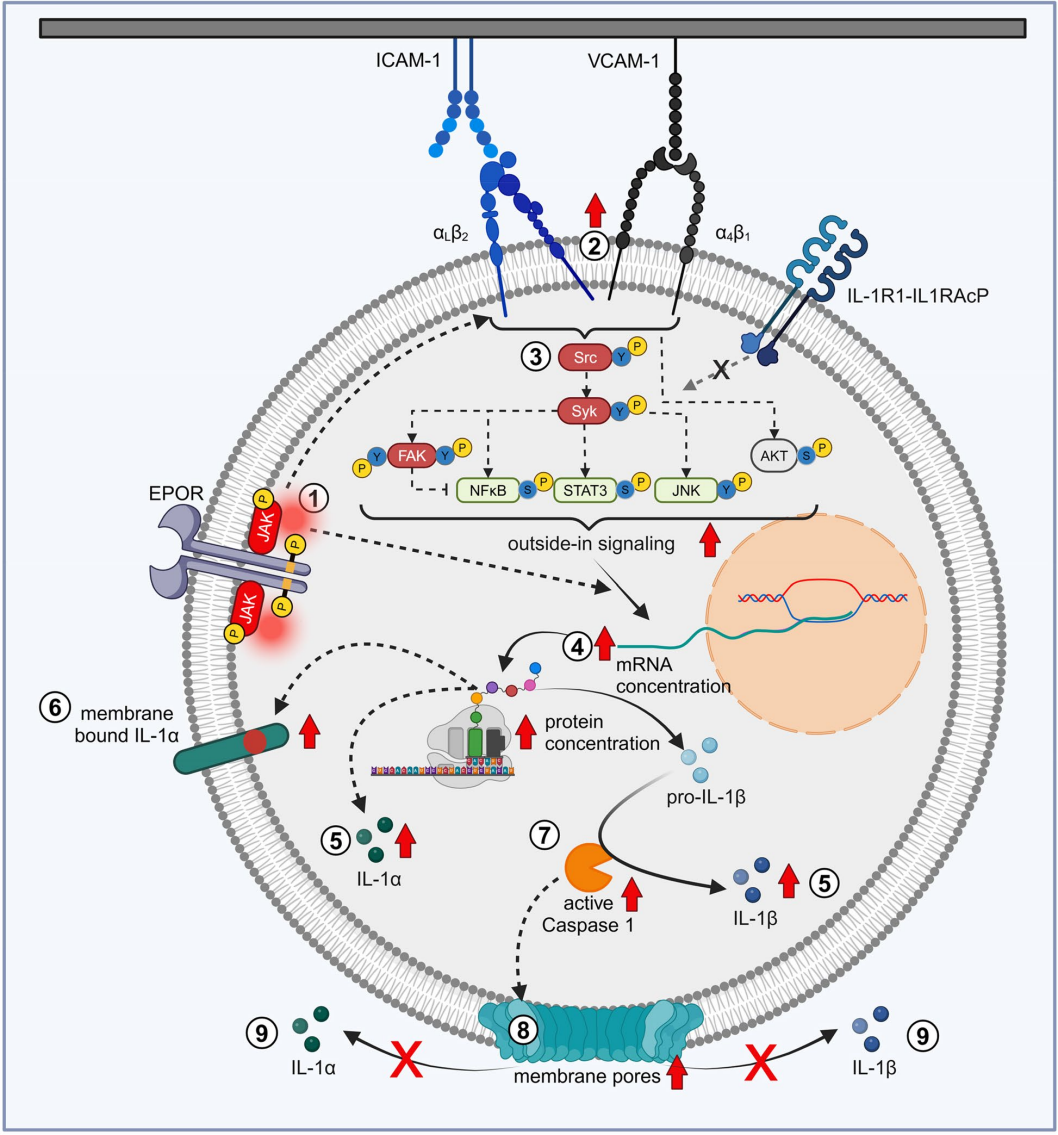
Cartoon: Signaling pathways and IL-1 protein processing in 32D JAK2-VF cells upon adhesion to VCAM-1/ICAM-1. ① Mutated JAK2 (JAK2-VF, shown as erythropoietin receptor (EPOR)-associated) induces ② constitutively active β1/β2-integrin conformation, which in turn increases adhesion to VCAM-1 and ICAM-1. ③ Upon binding to VCAM-1/ICAM-1, 32D JAK2-VF cells exhibit activated outside-in signaling, resulting in the phosphorylation of SRC, SYK, FAK, STAT3, NFκB, JNK, and AKT. FAK, SYK, NFκB and PI3K/AKT are key players in the induction of pro-inflammatory cytokine mRNA expression (*Il1a, Il1b, Il6, Tnf, Cxcl10*) upon VCAM-1/ICAM-1 stimulation ④. VCAM-1/ICAM-1 stimulation also induces several key biological processes, including ⑤ an increase in intracellular mature IL-1α and IL-1β protein concentrations, ⑥ the enhancement in membrane-bound IL-1α expression, and ⑦ elevation in caspase 1 activity, which is a critical step in the maturation of IL-1β. The presence of JAK2-VF promotes the expression of IL-1α and of IL-1β at both mRNA and protein levels, as well as the surface expression of IL-1α and activation of caspase 1. ⑧ Despite VCAM-1/ICAM-1 stimulation-induced pore formation (pyroptosis), ⑨ no secretion of IL-1 proteins is detectable (Created in BioRender. Haage, R. (2025) https://BioRender.com/v36d272)

It is clear that the majority of pro-IL-1β maturation occurs as a result of active caspase-1 [40]. We have shown for the first time that VCAM-1/ICAM-1 stimulation activates caspase 1, confirming successful pro-IL-1β maturation during stimulation in 32D JAK2 cells. NFκB is a key inducer of NLRP3 [106], pro-caspase 1 [107] and pro-IL-1β [91], which are essential for IL-1β maturation. For inflammasome assembling and activation, SYK-mediated phosphorylation of ASC (adaptor protein of the inflammasome) [96] and JNK-mediated phosphorylation of NLRP3 protein [108] are required. NFκB [91, 106, 107], SYK [96] and JNK [108] are all part of the inflammasome activation cascade and are activated upon integrin outside-in signaling in 32D JAK2 cells. This suggests that they are involved in the integrin-mediated caspase 1 activation demonstrated in our study. The secretion of mature IL-1α and IL-1β is typically mediated by pyroptosis in a cell type specific, stimulus specific and microenvironment specific manner [103, 109]. Our analysis employing live-dead staining combined with measurements of active caspase 1 definitively showed increased pyroptosis upon VCAM-1/ICAM-1 stimulation. However, we did not detect IL-1β secretion upon VCAM-1/ICAM-1 stimulation, neither in 32D cells nor in primary granulocytes isolated from JAK2-VF knock-in mice. This argues for the need for an additional stimulus for successful IL-1β secretion, which is absent in the in vitro cell culture. The cartoon depicted in Fig. 7 summarizes our results obtained in 32D JAK2-VF cells.

Granulocytes are important mediators of inflammation in JAK2-VF-positive MPN [13, 59, 78] and represent 25–75% of the leukocytes in the blood [110, 111]. Therefore, we investigated adhesion-induced mRNA cytokine expression in primary granulocytes isolated from *Vav1-Cre x Jak2*^+*/*+^ and *Vav1-Cre x Jak2*^*VF/*+^ mice upon VCAM-1/ICAM-1 stimulation. RNAseq results showed that VCAM-1/ICAM-1 stimulation clearly induced an overall increase in pro-inflammatory mRNAs. However, in contrast to 32D cells, a JAK2-VF-dependent increase of VCAM-1/ICAM-1-induced mRNA expression was not observed. In view of the pathophysiology of MPN, this points to the involvement of both *Jak2*^+*/*+^ and *Jak2*^*VF/*+^ cells in the development of the pro-inflammatory situation in JAK2-VF-positive disease. This is consistent with the conclusions of Kleppe et al. [112]. However, Rai et al., described JAK2-VF-positive hematopoietic cells as a primary source of inflammation [28]. In MPN patients, epigenetic dysregulation has been reported but is not specifically associated with JAK2-VF [81]. Consistent with this, we did not detect JAK2-VF specific differences in DNA methylation patterns of the pro-inflammatory genes investigated in primary granulocytes.

## Conclusion

In conclusion, we show that integrin stimulation via the adhesion molecules VCAM-1/ICAM-1 activates canonical integrin outside-in signaling in JAK2-V617F-mutated and non-mutated mouse hematopoietic cells. This includes elevated phosphorylation levels of Syk (Tyr519/520) and of JNK (Tyr183/185). Activated integrin outside-in signaling serves as a molecular link in up-regulation of a variety of pro-inflammatory cytokine mRNA levels including IL-1α and IL-1β. In 32D JAK2/EPOR cells, this also resulted in elevated IL-1α and IL-1β protein levels, whereas in granulocytes up-regulation of IL-1α and IL-1β at a protein level was not detectable. However, blocking of integrin binding to VCAM-1/ICAM-1 in vivo significantly reduced elevated IL-1α blood levels in JAK2-VF knock-in mice. These results point to a potentially important role of integrins in regulation of inflammatory cytokines in JAK2-V617F positive hematopoietic cells.

## Supplementary Information

Additional file 1. Analysis of fluorescence microscopy images. Detailed description of cell profiler analysis of fluorescence microscopy images.

Additional file 2. Supplementary Tables. Supplementary Tables 1–3 describeinhibitors and activators,Western blot antibodies andprimer sequences for mRNA quantification. Supplementary Table 4 describes the fluorescence microscopy system used.

Additional file 3. Supplementary Figs. 1–6 show additional results:mRNA expression upon IL-1α and VCAM-1/ICAM-1 stimulation in 32D JAK2-WT/EPOR, 32D JAK2-VF/EPOR cells,VCAM-1/ICAM-1 induced mRNA expression of inflammatory cytokines in lineage-negative hematopoietic cells, monocytes and B-cells.morphology of 32D JAK2-WT/EPOR and 32D JAK2-VF/EPOR cells upon VCAM-1/ICAM-1 stimulation,percentage caspase 1 active and pyroptosis-positive32D JAK2-WT/EPOR and 32D JAK2-VF/EPOR cells upon VCAM-1/ICAM-1 stimulation,principal component analysisof RNA-sequencing of granulocytes isolated from *Vav1-Cre x Jak2*^+*/*+^ and *Vav1-Cre x Jak2*^*VF/*+^ mice upon VCAM-1/ICAM-1 stimulation,Il-1α-induced migration of total bone marrow cells of *Vav1-Cre x Jak2*^+*/*+^ and *Vav1-Cre x Jak2*^*VF/*+^ mice.

Additional file 4.

## Data Availability

The datasets used and analyzed during the current study are available from the corresponding author on a reasonable request.

## Abbreviations

CalDAG-GEFI: Calcium-and-DAG-regulated guanine exchange factor-1
EPOR: Erythropoietin receptor
ET: Essential thrombocythemia
FAK: Focal adhesion kinase
FDR: False discovery rate
FLICA: Fluorescent labeled inhibitor of caspases assay
PLCγ: Phospholipase C gamma
GSEA: Gene set enrichment analysis
HU: Hydroxyurea
JNK: C-Jun-N-terminal kinase
MFI: Mean fluorescence intensity
MPN: Myeloproliferative neoplasms
NES: Normalized enrichment score
NFκB: Nuclear factor kappa B
PCA: Principal component analysis
Pic: Piceatannol
PI3K: Phosphatidylinositol-3 kinase
PMF: Primary myelofibrosis
PV: Polycythemia vera
RNAseq: RNA sequencing
Rux: Ruxolitinib
SYK: Spleen tyrosine kinase
WBM: Whole bone marrow
Wort: Wortmannin
