# Supplementary material for: Activation of integrin signaling up-regulates pro-inflammatory cytokines in JAK2-V617F positive hematopoietic cells
Source: Cell Commun Signal. 2025 Aug 11;23:368. doi: 10.1186/s12964-025-02358-x (PMC12337553; doi:10.1186/s12964-025-02358-x)
Supplement: Supplementary file 1 — Additional file 1. Analysis of fluorescence microscopy images. Detailed description of cell profiler analysis of fluorescence microscopy images. [file 12964_2025_2358_MOESM1_ESM.pdf]

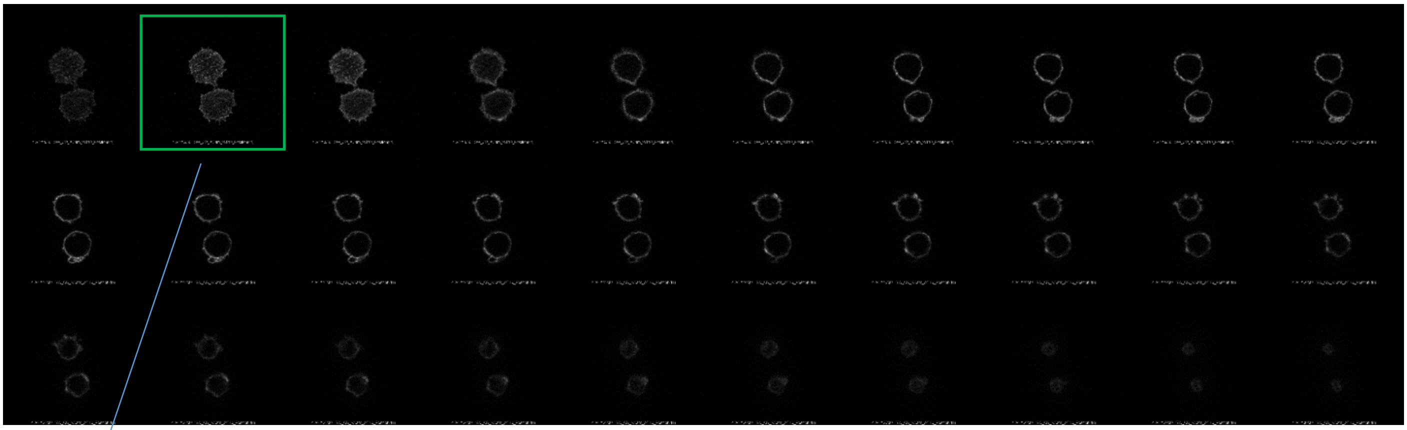

manual selection of the first in focus plane

CellProfiler™
cell image analysis software

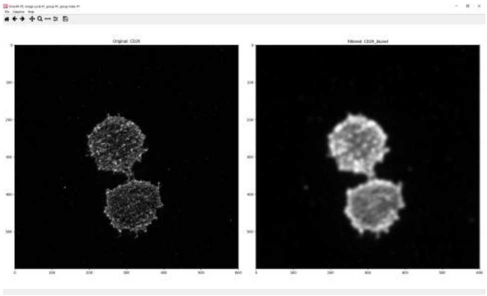

Step: Blur
- To fuse neighbouring objects (to identify cell bodies)
- Remove background

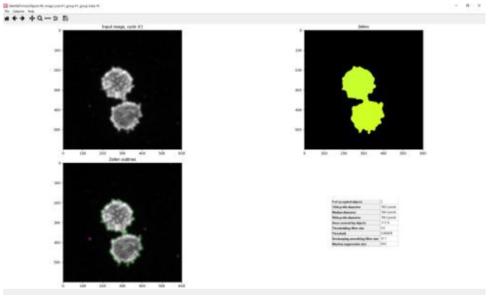

Step: identify cells
- Filter by size (i.e. remove smaller debris)
- Filter objects touching the edges of the image

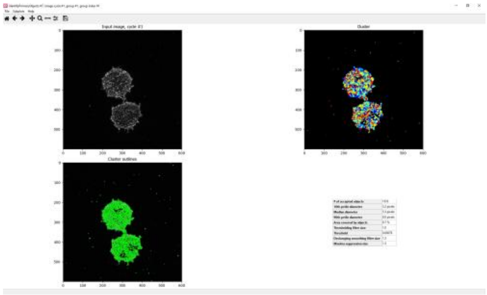

Step: identify cluster
- Filter by size
- declumping fused clusters

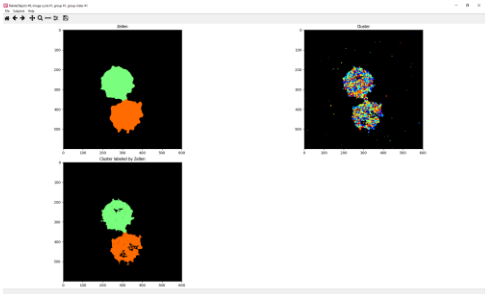

Step: relate clusters to identified cells
- to measure clusters per cell

Step: Export data

CellProfiler™
cell image analysis software

Segmentation quality assurance

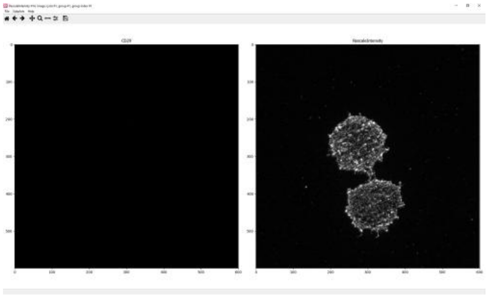

Step: rescale image intensity

- preparation for a later merged image

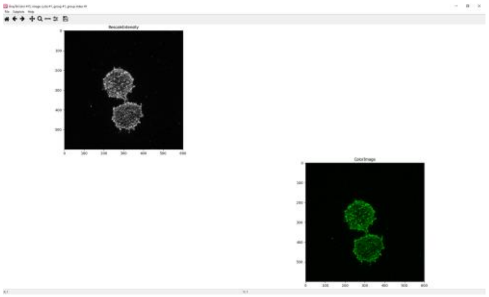

Step: apply green look up table

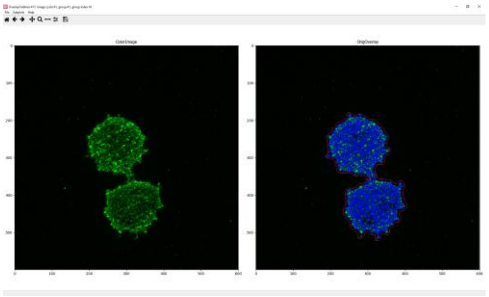

Step: superimpose object borders

- blue cluster
- red cell borders

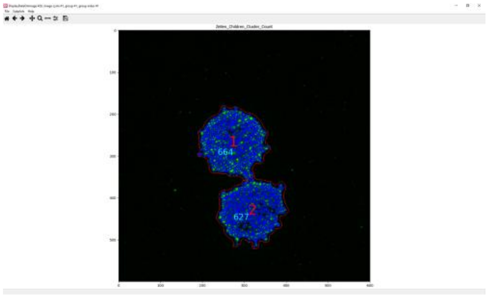

Step: superimpose data on image
and save file into the results foler

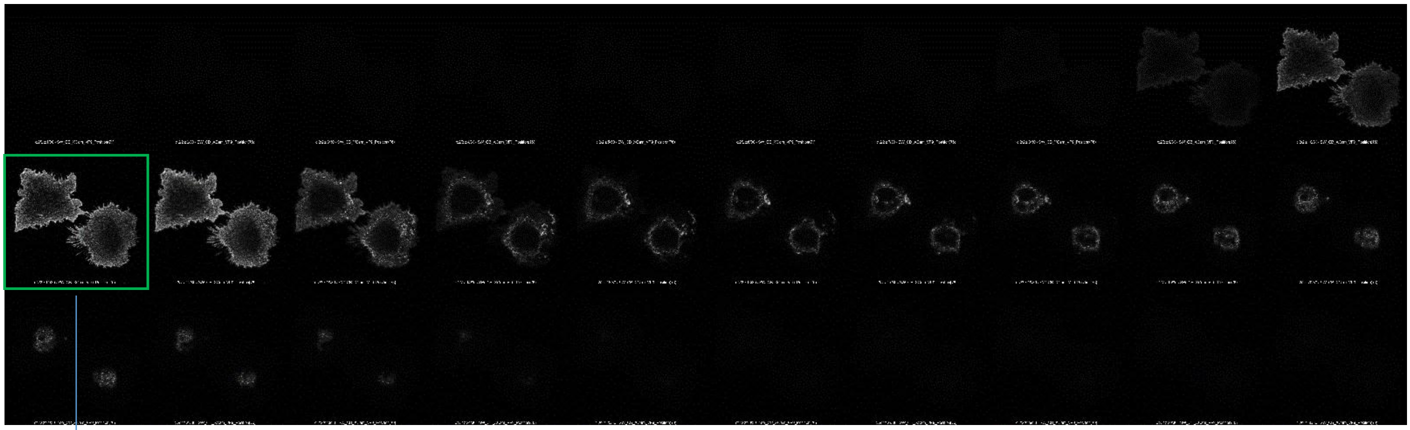

manual selection of the first in focus plane

CellProfiler™
cell image analysis software

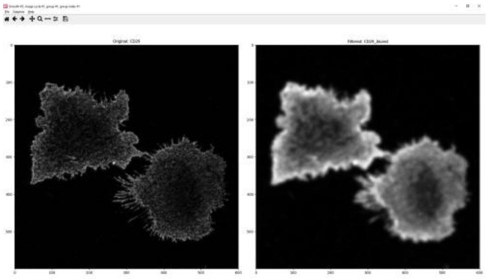

Step: Blur
- To fuse neighbouring objects (to identify cell bodies)
- Remove background

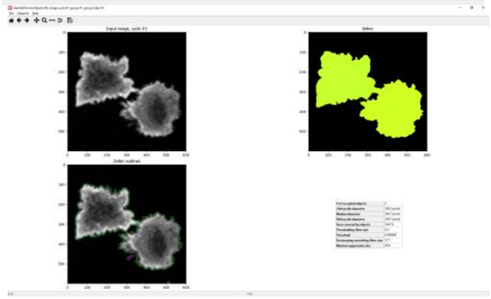

Step: identify cells
- Filter by size (i.e. remove smaller debris)
- Filter objects touching the edges of the image

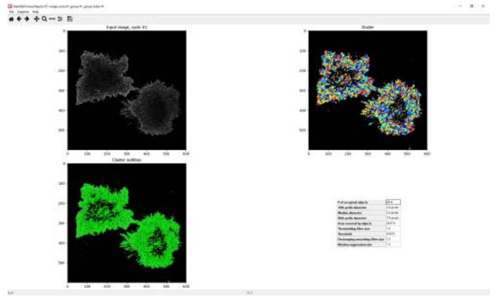

Step: identify cluster
- Filter by size
- declumping fused clusters

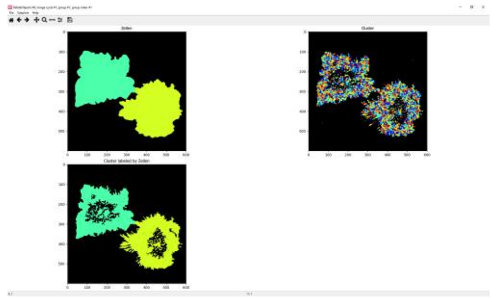

Step: relate clusters to identified cells
- to measure clusters per cell

Step: Export data

Segmentation quality assurance

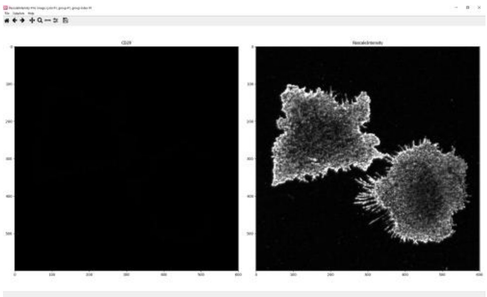

Step: rescale image intensity
- preparation for a later merged image

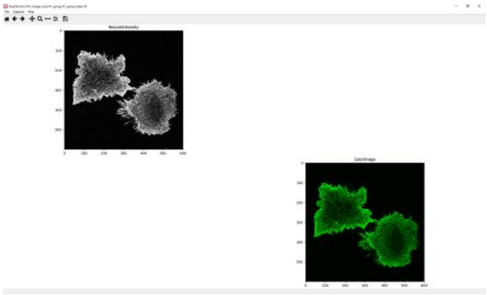

Step: apply green look up table

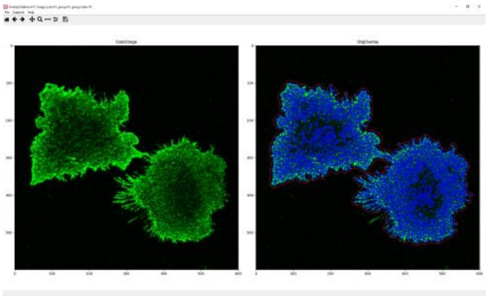

Step: superimpose object borders
- blue cluster
- red cell borders

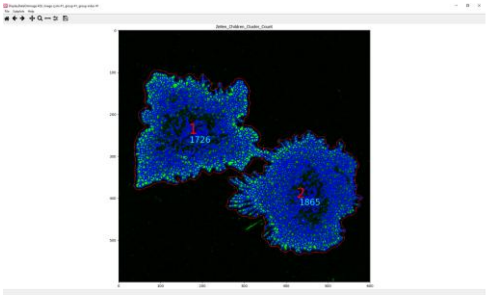

Step: superimpose data on image
and save file into the results foler
