# Supplementary material for: Activation of integrin signaling up-regulates pro-inflammatory cytokines in JAK2-V617F positive hematopoietic cells
Source: Cell Commun Signal. 2025 Aug 11;23:368. doi: 10.1186/s12964-025-02358-x (PMC12337553; doi:10.1186/s12964-025-02358-x)
Supplement: Supplementary file 2 — Additional file 2. Supplementary Tables. Supplementary Tables 1–3 describeinhibitors and activators,Western blot antibodies andprimer sequences for mRNA quantification. Supplementary Table 4 describes the fluorescence microscopy system used. [file 12964_2025_2358_MOESM2_ESM.pdf]

Additional File 2

Supplementary Table 1: Signaling molecule inhibitors and activators used for characterization of VCAM-1/ICAM-1-induced outside-in signaling.

inhibitor/activator	company	Cat-Nr.	concentration
**Adenosine 5′-triphosphate disodium salt hydrate (ATP)**	Sigma Aldrich	A6419-1g	3 mM
**Hydroxyurea (HU)**	Sigma	H8627-1G	0.5 mM
**recombinant IL-1RA**	Biolegend	769704	
**JSH-23**	Selleckchem	S7351	50 µM
**Nigericin**	AdipoGen	AG-CN2-0020-M005	5 µM
**Piceatannol (Pic)**	Selleckchem	S3026	30 µM
**PP2**	Selleckchem	S7008	5 µM
**Ruxolitinib (RUX)**	Selleckchem	S1378	2 µM
**Wortmannin (Wort)**	Selleckchem	S2758	50 nM
**Y15**	Selleckchem	S5321	25 µM

Supplementary Table 2: Western Blot antibodies used for characterization of outside-in signaling

antibody	epitope	size in kDa	organism of origin	dilution	company	Cat-Nr.
**primary antibodies**						
anti-pFAK (Tyr397)	Tyr397	125	rabbit	1:500	Cell Signaling	9330T
Anti-pFAK (Tyr577)	Tyr577	125	rabbit	1:500	Cell Signaling	9330T
anti-FAK	total protein	125	rabbit	1:500	Cell Signaling	9330T
anti-pSTAT3	Ser727	79, 86	mouse	1:500	Cell Signaling	9134S
anti-STAT3	residues in close proximity of AS Gln692	79, 86	mouse	1:500	Cell Signaling	9139S
anti-pSyk	Tyr519/520	72	rabbit	1:1000	Cell Signaling	2710S
anti-Syk	N-terminus	72	rabbit	1:1000	Cell Signaling	1235S
anti-pNFκB	Ser536	65	rabbit	1:1000	Cell Signaling	3033
anti-NFκB	residues in close proximity of AS Glu498	65	rabbit	1:500	Cell Signaling	8242
anti-pSAPK/JNK	Thr183/ Tyr185	46, 54	rabbit	1:1000	Cell Signaling	9251
anti-SAPK/JNK	total protein	46, 54	rabbit	1:1000	Cell Signaling	9252
anti-GAPDH	total protein	36	mouse	1:2500	Meridian Life Science	H86504M
**secondary antibodies**						
anti-mouse-IgGκ-HRP	IgGκ	-	recombinant	1:2000 (in case of GAPDH 1:4000)	Santa Cruz	sc2005
anti-rabbit-IgG-HRP	IgG	-	goat	1:2000	Cell Signaling	7074S

Supplementary Table 3: Primer sequences used for qPCR analyses to detect pro-inflammatory cytokine mRNAs.

Gene	orientation	Primer sequence
Il1a	For	5’-TCTCAGATTCACAACTGTTCGTG-3’
	Rev	5’-AGAAAATGAGGTCGGTCTCACTA-3’
Il1b	For	5’-TTGACGGACCCCAAAAGATG-3’
	Rev	5’-AGAAGGTGCTCATGTCCTCA-3’
Il6	For	5’-CTGCAAGAGACTTCCATCCAG-3’
	Rev	5’-AGTGGTATAGACAGGTCTGTTGG-3’
Tnf	For	5’-GGTGCCTATGTCTCAGCCTCTT-3’
	Rev	5’-GCCATAGAACTGATGAGAGGGAG-3’
Cxcl10	For	5’-AGTGCTGCCGTCATTTTCTG-3’
	Rev	5’-ATTCTCACTGGCCCGTCAT-3’
Hprt	For	5’-GGACAGGACTGAAAGACTTG-3’
	Rev	5’-CGTTGACTGATCATTACAGTAGC-3’

Supplementary Table 4: Characterization of the fluorescence microscopy system used.

device	name	company
**microscope**	Zeiss Axiovert 200m	Carl Zeiss
**light source**	Zeiss Colibri2	Carl Zeiss
**structured lighting**	Zeiss Apotome2	Carl Zeiss
**filter set (FS)**	FS62 HE<br>EGFP ET filter set (AHF)<br>F49-470 470/40 ET bandpass<br>F48-495 beam splitter T 495 LPXR<br>F47-525 525/50 ET bandpass	Carl Zeiss
**camera**	AxioCam MRM	Carl Zeiss
**cross table**	motorized Marchhäuser cross table	Märzhaeuser Wetzlar GmbH & Co. KG
**lens**	Plan-Apochromat 63x/1.4 Oil	Carl Zeiss
**software**	AxioVision 4.8	Carl Zeiss
	CellProfiler 4.2.1	Broad Institute
