# Supplementary material for: Activation of integrin signaling up-regulates pro-inflammatory cytokines in JAK2-V617F positive hematopoietic cells
Source: Cell Commun Signal. 2025 Aug 11;23:368. doi: 10.1186/s12964-025-02358-x (PMC12337553; doi:10.1186/s12964-025-02358-x)
Supplement: Supplementary file 3 — Additional file 3. Supplementary Figs. 1–6 show additional results:mRNA expression upon IL-1α and VCAM-1/ICAM-1 stimulation in 32D JAK2-WT/EPOR, 32D JAK2-VF/EPOR cells,VCAM-1/ICAM-1 induced mRNA expression of inflammatory cytokines in lineage-negative hematopoietic cells, monocytes and B-cells.morphology of 32D JAK2-WT/EPOR and 32D JAK2-VF/EPOR cells upon VCAM-1/ICAM-1 stimulation,percentage caspase 1 active and pyroptosis-positive32D JAK2-WT/EPOR and 32D JAK2-VF/EPOR cells upon VCAM-1/ICAM-1 stimulation,principal component analysisof RNA-sequencing of granulocytes isolated from Vav1-Cre x Jak2+/+ and Vav1-Cre x Jak2VF/+ mice upon VCAM-1/ICAM-1 stimulation,Il-1α-induced migration of total bone marrow cells of Vav1-Cre x Jak2+/+ and Vav1-Cre x Jak2VF/+ mice. [file 12964_2025_2358_MOESM3_ESM.pdf]

Supplementary Figure 1

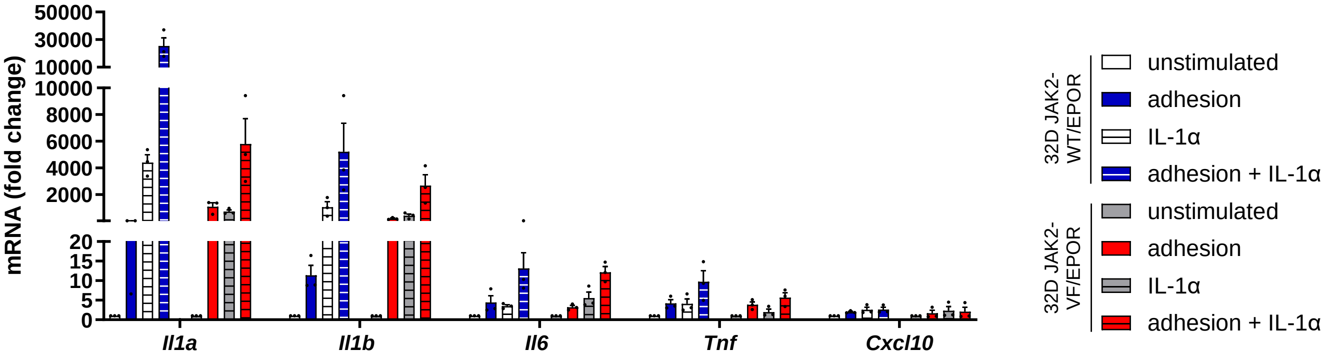

Supplementary Figure 1: IL-1α promotes adhesion-induced mRNA expression of inflammatory cytokines. mRNA cytokine expression (*Il1a, Il1b, Il6, Tnf, Cxcl10*) of 32D JAK2-WT/EPOR (n = 3) and 32D JAK2-VF/EPOR cells (n = 3) was monitored upon stimulation with VCAM-1/ICAM-1. Stimulation with BSA (“unstimulated”) was used as a control. Additionally, in conditions indicated, the cells were pre-incubated with recombinant IL-1α (10 ng/ml) for 24 h. In these cases, IL-1α (10 ng/ml) was also added during stimulation. 32D JAK2/EPOR cells were stimulated for 3 h prior to RNA isolation and qPCR analysis. Data are shown as fold change ($2^{-\Delta\Delta CT}$) as mean + SEM.

Supplementary Figure 2

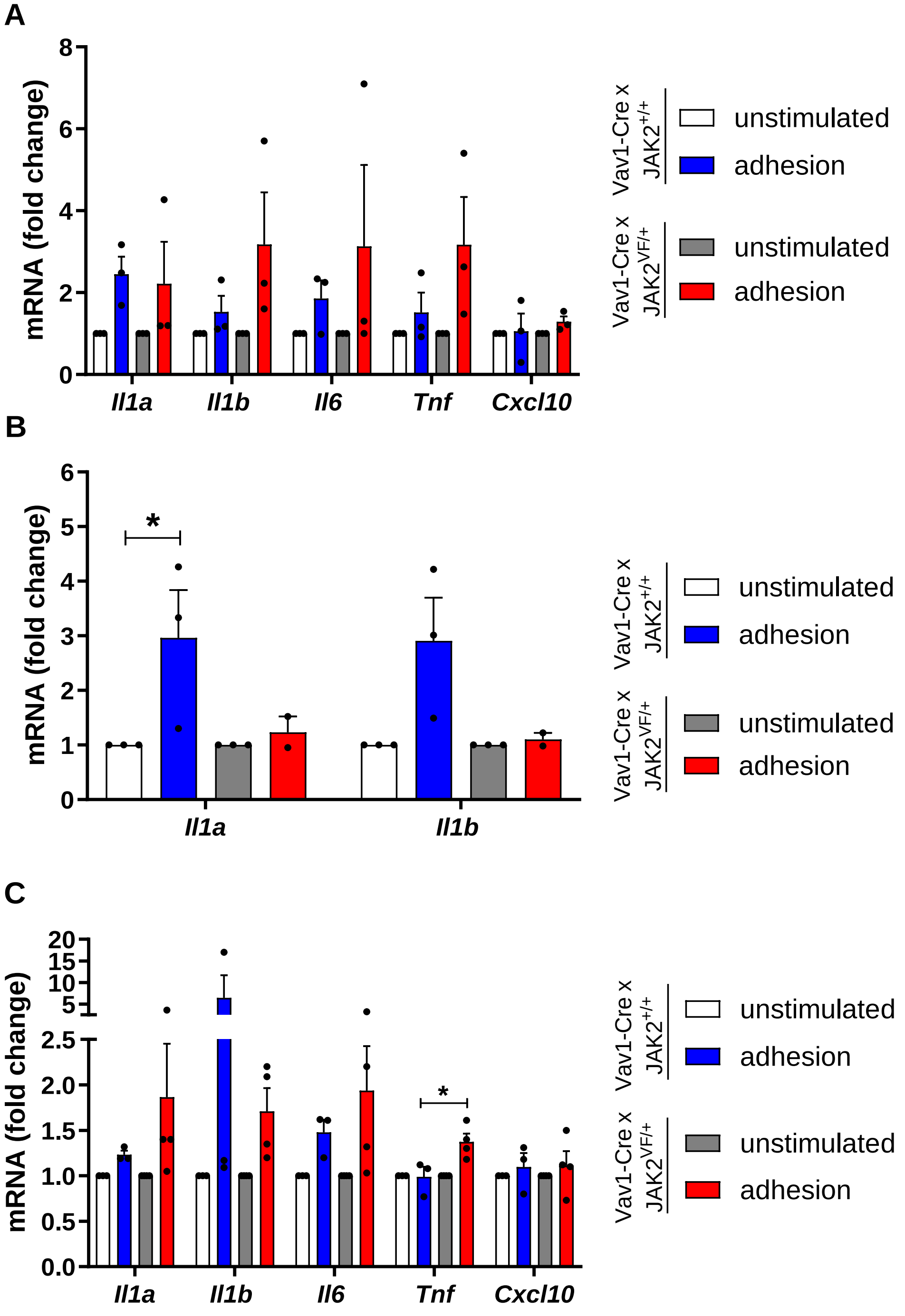

Supplementary Figure 3: VCAM-1/ICAM-1 stimulation induces mRNA expression of inflammatory cytokines in various hematopoietic cell populations. Cytokine mRNA expression upon VCAM-1/ICAM-1 stimulation (1 h) using isolated **(A)** lineage-negative cells (including hematopoietic stem and progenitor cells), **(B)** monocytes and **(C)** B cells of Vav1-Cre x $Jak2^{+/+}$ (n=7) and Vav1-Cre x $Jak2^{VF/+}$ mice (n=7) is shown as fold change compared to the BSA control (“unstimulated”). Data are shown as fold change ($2^{-\Delta\Delta CT}$) with mean + SEM. Mixed-effects analysis with P*<0.05.

Supplementary Figure 3

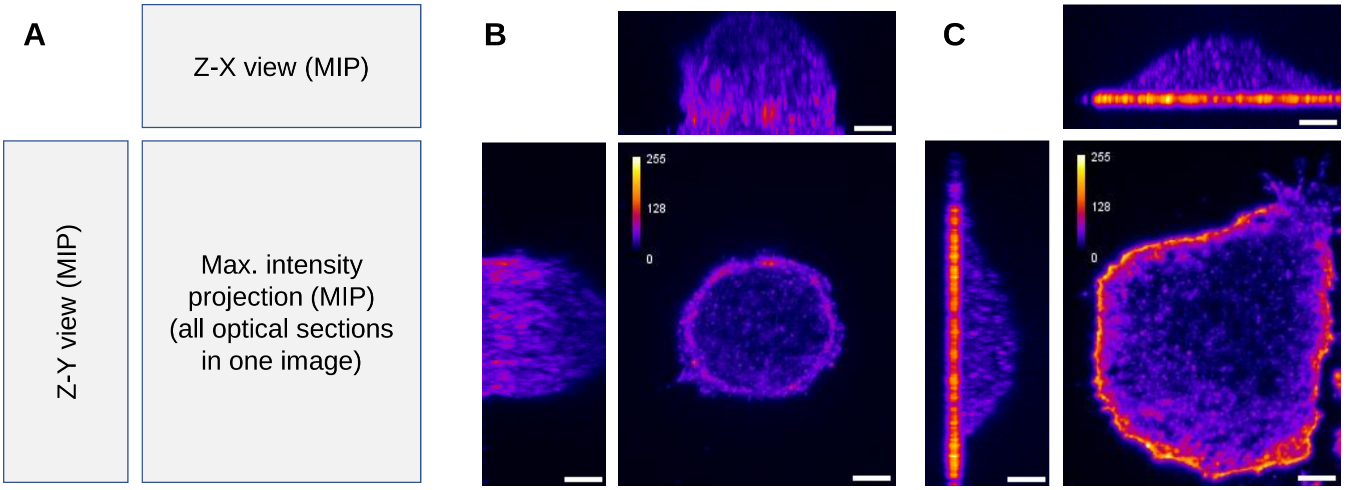

Supplementary Figure 3: Analysis of β1 integrin clustering of 32D JAK2-WT cells versus 32D JAK2-VF cells upon stimulation with immobilized VCAM-1/ICAM-1. (A) Maximum intensity projection of fluorescent microscopy of β1-integrins (antibody used: anti-CD29-AlexaFluor488® antibody (clone HMβ1-1, Biolegend)) upon adhesion on immobilized VCAM-1/ICAM-1 is shown as scheme and for one representative **(B)** 32D JAK2-WT/EPOR and **(C)** and 32D JAK2-VF/EPOR cell. Representative fluorescence microscopy images are shown. The brightness of these images has been normalized to the highest value and is presented in false colors, ranging from dark blue (low fluorescence intensity) to bright yellow (high fluorescence intensity). The scale bar represents 3 µm.

Supplementary Figure 4

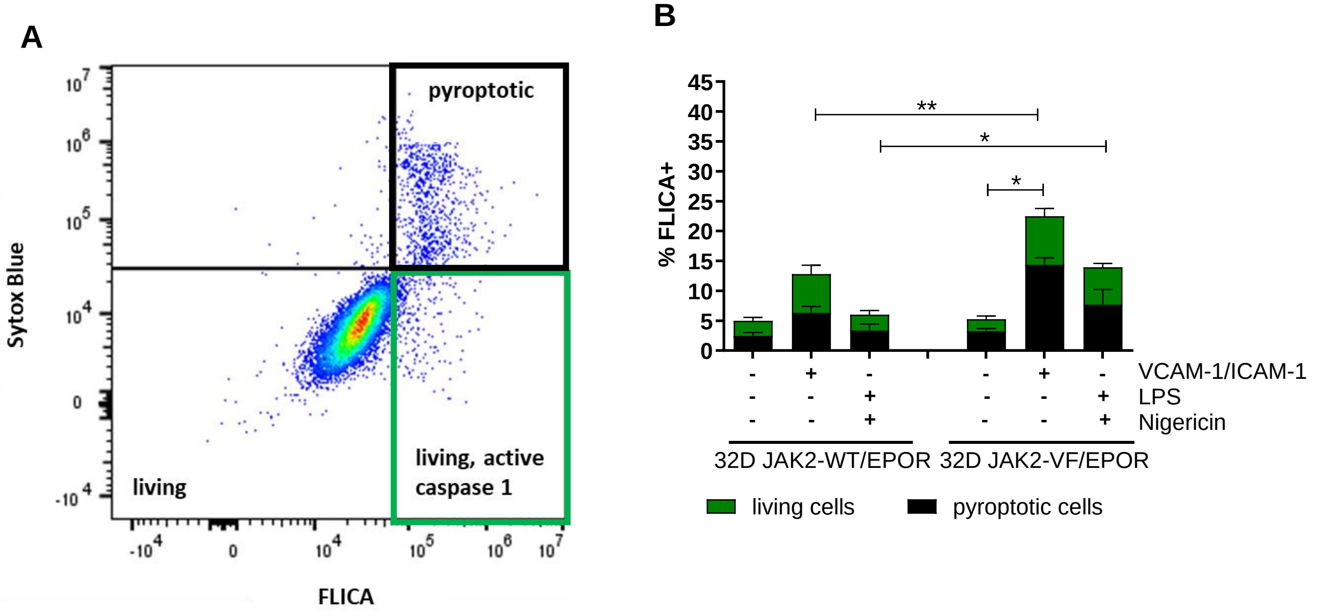

Supplementary Figure 4: VCAM-1/ICAM-1 stimulation increases the fraction of active caspase 1 positive and pyroptotic cells. (A) Representative gating strategy of FLICA assay. **(B)** 32D JAK2-WT/EPOR (left; n = 4) and 32D JAK2-VF/EPOR cells (right; n = 4) were stimulated for 6 h with immobilized VCAM-1/ICAM-1 or 100 ng/ml LPS plus 5 µM Nigericin. Stimulation using BSA served as controls. FLICA probe (FAM-YVAD-FMK) was added 1 h before stimulation was terminated. Cells were harvested and analyzed by flow cytometry (Cytek® Northern Lights™)**.** Cells which are single FLICA positive contain active caspase 1, but are alive cells without pore formation (A: lower right). Double positive cells ($FLICA^+$, $SytoxBlue^+$) represent pyroptotic cells with active caspase 1 and pore formation (A: upper right). (**B**) Quantification of FLICA positive cells shown as percentage (mean + SEM) divided by alive cells (FLICA-positive, SytoxBlue-negative; green) and pyroptotic cells ($FLICA^+$, $SytoxBlue^+$; black). Data are shown as mean + SEM. Ordinary one-way ANOVA (Holm-Šídák's multiple comparisons test) with $P^* < 0.05$, $P^{**} < 0.01$.

Supplementary Figure 5

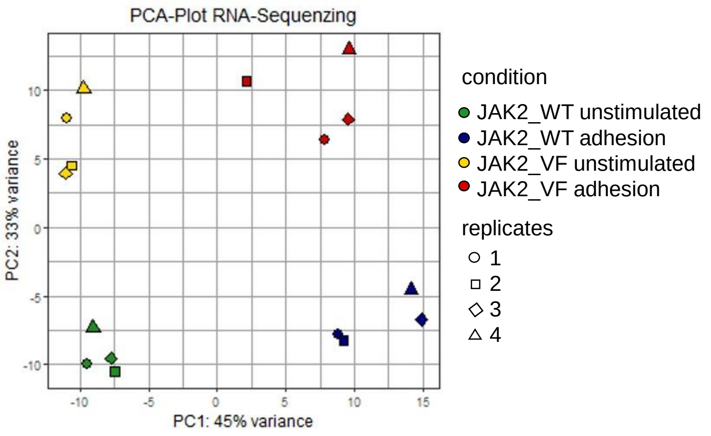

Supplementary Figure 5: Principal component analysis (PCA) of the granulocyte RNA-sequencing. *Vav1-Cre x Jak2*$^{+/+}$ (JAK2_WT; n = 4) and *Vav1-Cre x Jak2*$^{VF/+}$ (JAK2_VF; n = 4) granulocytes were stimulated with VCAM-1/ICAM-1 (adhesion) or BSA (unstimulated) for 1 h. RNA was isolated and sequenced. Principal component analysis (PCA) was performed using R Studios. The replicates are represented by geometric shapes, the conditions in colors.

Supplementary Figure 6

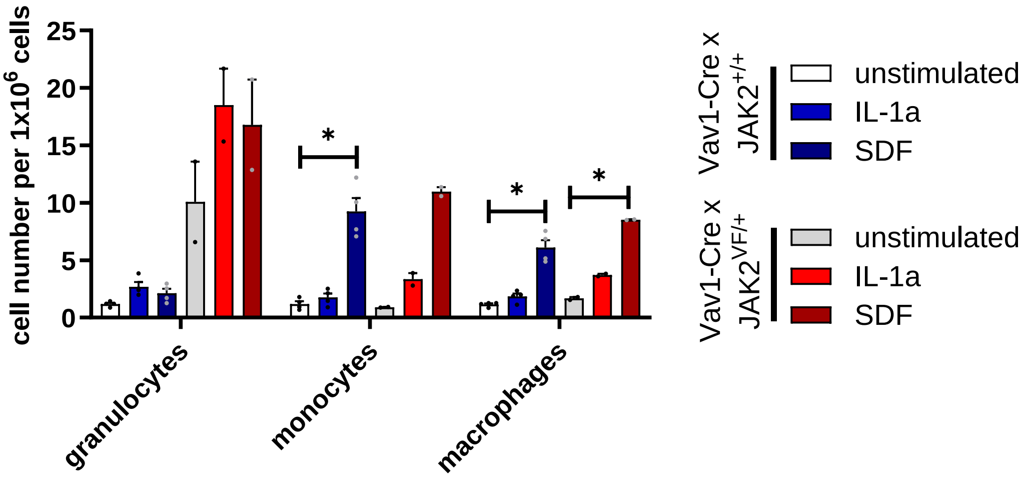

Supplementary Figure 6: IL-1α promotes migration of JAK2-V617F mutated granulocytes, monocytes and macrophages of *Vav1-Cre x Jak2$^{VF/+}$* mice. Total bone marrow cells were isolated of *Vav1-Cre x Jak2$^{+/+}$* (n = 4) and *Vav1-Cre x Jak2$^{VF/+}$* (n = 2) mice. A migration assay was performed using a transwell system (Corning® 6.5 mm Transwell® with 5.0 µm Pore Polycarbonate Membrane Insert). Recombinant IL-1α (10 ng/ml) was added in the lower well. SDF-α (300 ng/ml) was used as positive control. RPMI supplemented with 5 % FCS was used. Three times 100,000 cells were seeded for each condition. After 6 h, migrated cells in the lower wells were harvested, pooled and analyzed by flow cytometry. Data of monocytes ($CD11b^+$, $Ly6C^+$, $Ly6G^-$), granulocytes ($CD11b^+$, $Ly6C^{low}$, $Ly6G^+$) and macrophages ($CD11b^+$, $F4/80^+$) are shown as cell number per 1 x 10^6 cells, which were migrated. Data are shown as mean + SEM. Mixed-effect analysis (Šídák's multiple comparisons test) with P*<0.05.
