# Supplementary material for: Activation of integrin signaling up-regulates pro-inflammatory cytokines in JAK2-V617F positive hematopoietic cells
Source: Cell Commun Signal. 2025 Aug 11;23:368. doi: 10.1186/s12964-025-02358-x (PMC12337553; doi:10.1186/s12964-025-02358-x)
Supplement: Supplementary file 4 — Additional file 4. [file 12964_2025_2358_MOESM4_ESM.pdf]

To Figure 2A left

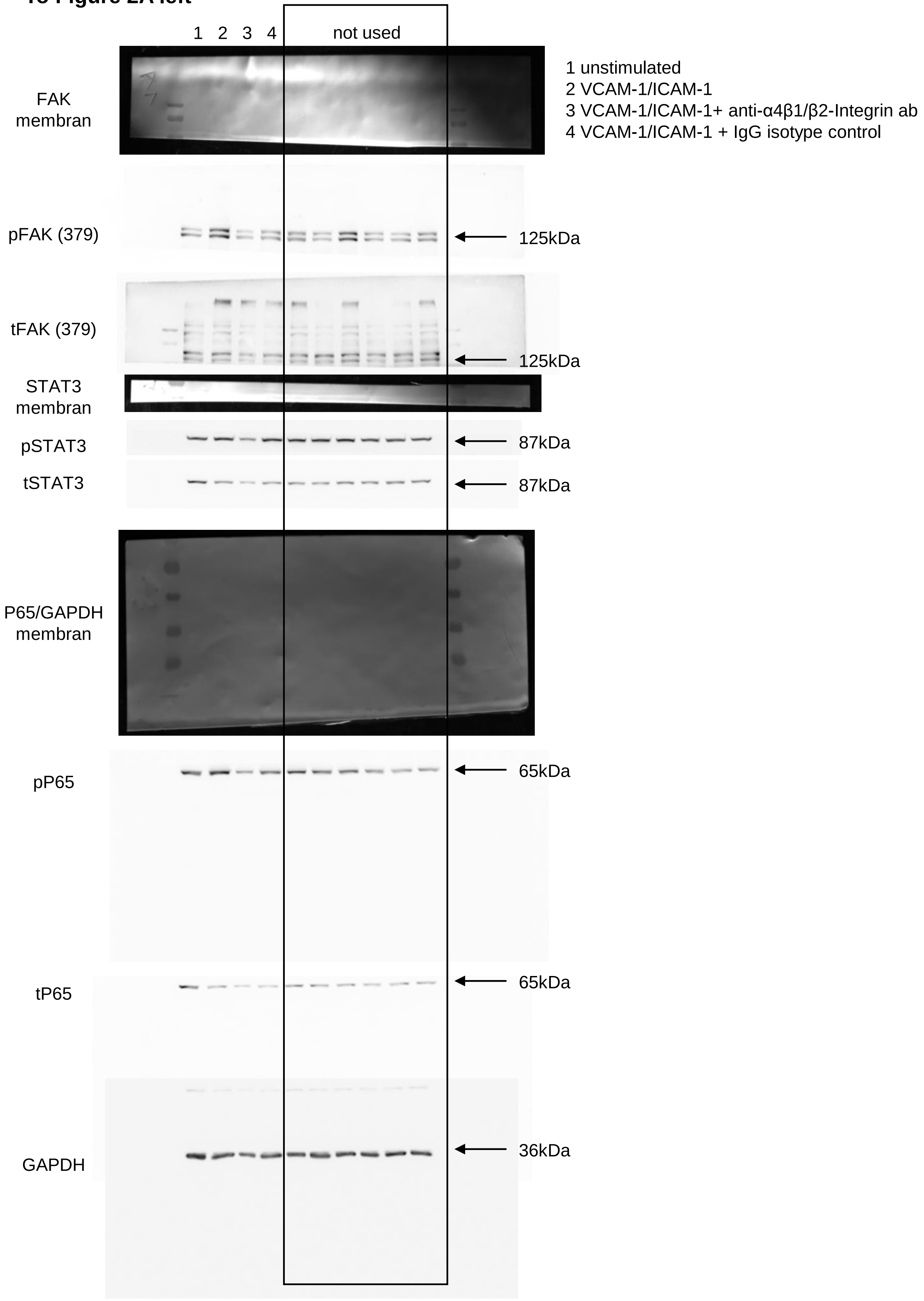

To Figure 2A right

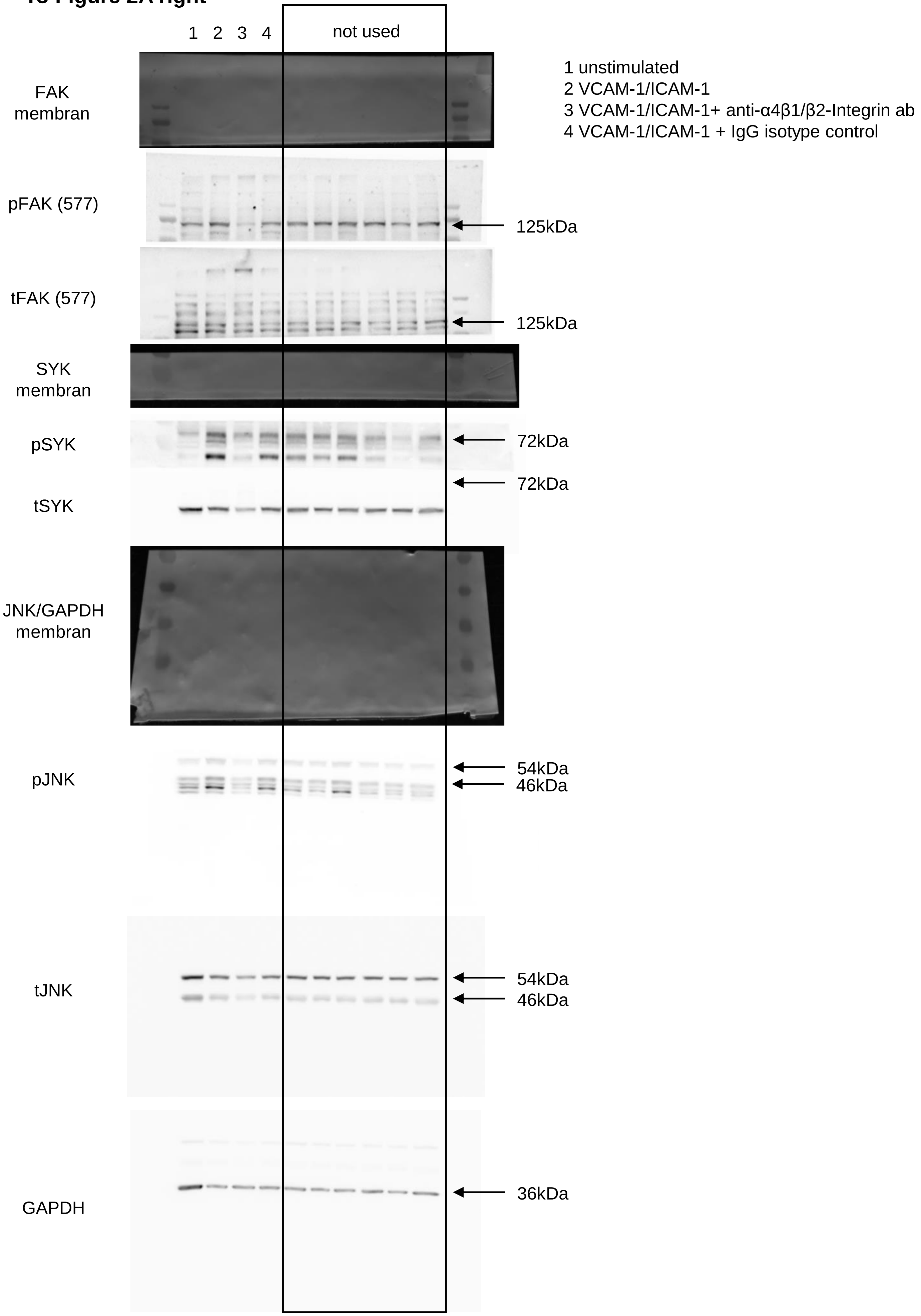

To Figure 2D

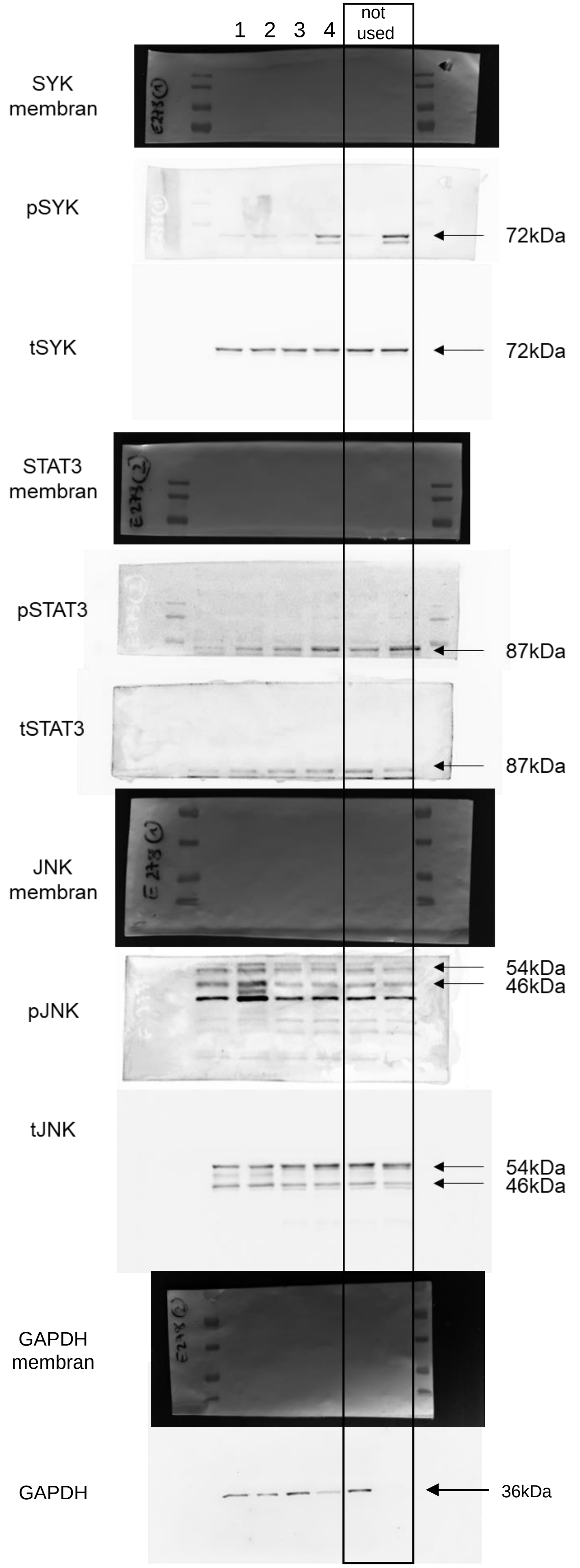

1 unstimulated *Vav1-Cre x JAK2*$^{+/+}$
2 VCAM-1/ICAM-1 *Vav1-Cre x JAK2*$^{+/+}$

3 unstimulated *Vav1-Cre x JAK2*$^{VF/+}$
4 VCAM-1/ICAM-1*Vav1-Cre x JAK2*$^{VF/+}$
